# Parsing the Q-Markers of Baoyin Jian to Treat Abnormal Uterine Bleeding by High-Throughput Chinmedomics Strategy

**DOI:** 10.3390/ph16050719

**Published:** 2023-05-09

**Authors:** Qiuhan Li, Junling Ren, Le Yang, Hui Sun, Xiwu Zhang, Guangli Yan, Ying Han, Xijun Wang

**Affiliations:** 1 National Chinmedomics Research Center, National TCM Key Laboratory of Serum Pharmacochemistry, Metabolomics Laboratory, Department of Pharmaceutical Analysis, Heilongjiang University of Chinese Medicine, Heping Road 24, Harbin 150040, China; 15846507205@163.com (Q.L.); junlingren@126.com (J.R.); zxw506zxw@163.com (X.Z.); gancaosuan@163.com (G.Y.); hanying314@sina.com (Y.H.); 2 State Key Laboratory of Quality Research in Chinese Medicine, Macau University of Science and Technology, Avenida Wai Long, Taipa 999078, Macau; 3 State Key Laboratory of Dampness Syndrome, The Second Affiliated Hospital Guangzhou University of Chinese Medicine, Dade Road 111, Guangzhou 510120, China; leyang92gzy@sina.com

**Keywords:** Baoyin Jian, abnormal uterine bleeding, Chinmedomics, Q-marker, quality control

## Abstract

Abnormal uterine bleeding (AUB) is a common and frequently occurring disease in gynecology, seriously threatening women’s health. Baoyin Jian (BYJ) is a classical prescription for treating AUB. However, the lack of quality control standards of BYJ for AUB have limited the development and applications of BYJ. This experiment aims to explore the mechanism of action and screen the quality markers (Q-markers) of BYJ against AUB through the Chinmedomics strategy to improve the quality standards of Chinese medicine and provide scientific basis for its further development. BYJ has hemostatic effects in rats, as well as the ability to regulate the coagulation system following incomplete medical abortion. According to the results of histopathology, biochemical indexes and urine metabolomics, a total of 32 biomarkers of ABU in rats were identified, 16 of which can be significantly regulated by BYJ. Using traditional Chinese medicine (TCM) serum pharmacochemistry technology, 59 effective components were detected *in vivo*, of which 13 were highly correlated with efficacy, and 9 components, namely catalpol, rehmannioside D, paeoniflorin, berberine, phellodendrine, baicalin, asperosaponinVI, liquiritin, and glycyrrhizic acid, were screened out as the Q-markers of BYJ based on the “Five Principles” of Q-markers. In sum, BYJ can effectively alleviate abnormal bleeding symptoms and metabolic abnormalities in AUB rats. The study shows that Chinmedomics is an effective tool for screening Q-markers and provides scientific support for the further development and clinical use of BYJ.

## 1. Introduction

Abnormal uterine bleeding (AUB) is a common gynecological disease, usually manifesting in excessive menstruation, prolonged menstruation, and irregular vaginal bleeding, which seriously affects the work efficiency and quality of life of patients. Modern medicine holds that AUB is caused by neuroendocrine disorders regulated by the hypothalamic–pituitary–ovary axis (HPOA) [1]. At present, estrogen and progesterone drugs are often used in clinical treatment. Although hormone therapy has certain benefits, it has side effects such as weight gain, gastrointestinal reactions and increased liver burden, and AUB may recur after stopping taking the drugs [2]. Due to its complicated etiology and pathogenesis, and the limitations of existing therapeutic drugs, it is very important to seek a better treatment scheme to improve curative effects and reduce recurrence. In recent years, many clinical studies have shown that traditional Chinese medicine (TCM) has a good effect on AUB and a low recurrence rate, and is easily tolerated by patients [3].

Baoyin Jian (BYJ) is a classic prescription for the treatment of hemorrhagic disorders in gynecology. It originated in *Jingyue Quanshu*, written by Zhang Jingyue in the Ming Dynasty, and consists of Rehmanniae Radix; Rehmanniae Radix Praeparata; Paeoniae Radix Alba; Phellodendri Chinensis Cortex; Scutellariae Radix; Dipsaci Radix; Dioscoreae Rhizoma; and Glycyrrhizae Radlx et Rhizoma. It has the effects of nourishing yin deficiency, nourishing blood, and stopping bleeding [4]. In modern clinical practice, BYJ is mainly used to treat abnormal uterine bleeding, threatened abortion, menorrhagia, and other gynecological bleeding-related diseases [5]. While most of the studies on BYJ focused on clinical efficacy evaluation and identification of chemical components *in vitro*, there is a lack of research on quality markers (Q-Markers) related to clinical efficacy, which makes it difficult to investigate the basic characteristics of BYJ in the treatment of AUB and limits the assurance of its clinical efficacy, which also hinders the clinical promotion of BYJ [6].

The quality of TCM is the basis to ensure its clinical efficacy and safety. However, due to the complex components of TCM prescription, the existing quality control standards for Chinese medicines are not sufficient to objectively evaluate their quality, and it is difficult to guarantee the safety and efficacy of their clinical use [7]. The Q-marker concept, combined with the characteristics of TCM and its use in clinical practice, links efficacy, effective constituents, and quality, and improves the deficiencies of the existing quality standards of TCM. Chinmedomics refers to the discovery of biomarkers for TCM syndromes through metabolomics technology, the application of the TCM serum pharmacochemical method to analyze active ingredients in the body under the effective condition of TCM prescriptions, the correlation between these and biomarkers, and the selection of drug ingredients highly associated with biomarker trajectory changes. Thus, the theoretical system of biological evaluation of TCM efficacy, pharmacodynamic material basis, and mechanism of action was elucidated. Chinmedomics is an effective way to investigate the effectiveness, traceability, and compatibility of prescriptions of Q-markers. In recent years, Q-markers were successfully discovered in various single herbs and prescriptions, such as American ginseng, Yinchenhao Decoction, ZhibaiDihuang Pill and Wenxin Formula, etc., by employing Chinmedomics [8,9,10,11]. The successful application of Chinmedomics helps to comprehensively and accurately identify the Q-markers of TCM and provides a powerful means of quality control of TCM.

We aimed to elucidate the potential efficacy and mechanism of action of BYJ in the treatment of AUB and to identify its Q-markers, which can help improve the quality standards of Chinese medicine. In the present study, based on an AUB rat model, histopathological and biochemical indices were used to evaluate the efficacy of BYJ in the treatment of AUB. Then, under the guidance of Chinmedomics, we evaluated the changes to the overall metabolic profile of AUB rats after treatment with BYJ and investigated the active components closely related to the efficacy of BYJ based on the “Five Principles” of Q-markers to identify the potential Q-markers of BYJ for the treatment of AUB. This study provides technical guidance for the development and utilization of the famous classical formulae and the further development of quality control standards.

## 2. Results

### 2.1. Pharmacodynamic Evaluation of BYJ on AUB Rats

#### 2.1.1. Morphological and Pathological Analysis

**Figure 1:**
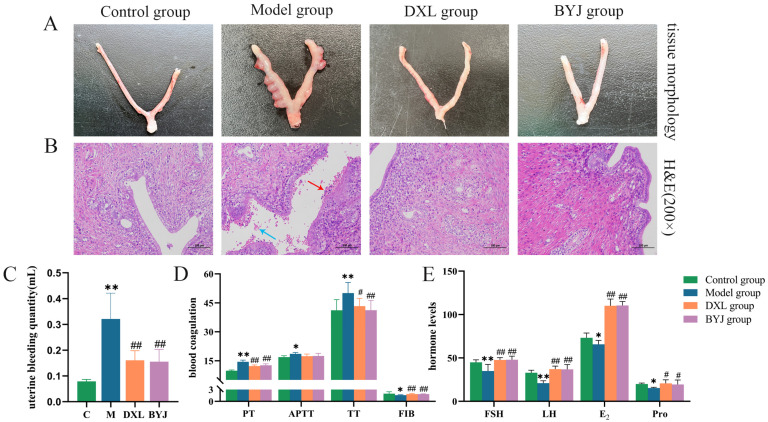
Pharmacodynamic evaluation of BYJ on AUB rats. (**A**) Macroscopic appearance of the uterus. (**B**) Histopathological examination (×200). (**C**) Uterine bleeding volume. (**D**) Coagulation function. (**E**) Hormone levels. Results are expressed as mean ± SD, *n* = 6. * *p* < 0.05, ** *p* < 0.01 compared with control group. ^#^ *p* < 0.05, ^##^ *p* < 0.01 compared with model group. Control group: normal non-pregnant female rat; model group: the AUB model of incomplete medical abortion in early pregnancy was established by taking mifepristone and misoprostol orally on the 7th day of pregnancy; DXL group: positive control group with Duanxueliu solution; BYJ group: treatment group with Baoyin Jian.

It was observed that the uterine morphology of normal non-pregnant rats was intact, with a smooth and ruddy surface. In contrast, the uterine tissues of the model group were enlarged, twisted, and dark red in color, the uterine cavity showed embryonic remnants and congestion, and the embryonic boundary was unclear, indicating incomplete abortion. The positive drug group (Duanxueliu solution, DXL) and treatment group (BYJ) showed no enlarged nodules, no visible residual necrotic tissue, and improved congestion. Macroscopically, the uterine tissues of these groups had returned to normal morphology (Figure 1A).

Hematoxylin and eosin (HE) staining results showed that the endometrial cells of the control group were intact and neatly arranged. However, in the model group, numerous endometrial cells were seen to be necrotic, with visible bleeding (blue arrows) and neutrophil infiltration (red arrows). It was confirmed the AUB model was successfully replicated. In the DXL and BYJ groups, there were no apparent residual endometrial cells or neutrophil infiltration, and no visible bleeding in the uterine cavity. These findings suggest that the damage to the uterine tissues was alleviated to varying degrees (Figure 1B).

#### 2.1.2. Uterine Bleeding Volume Evaluation

Compared with the control group, uterine bleeding volume was increased significantly in the model group (*p* < 0.01), indicating the successful replication of the model. Compared with the model group, the DXL and the BYJ group showed a significant decrease in uterine bleeding (*p* < 0.05), indicating that BYJ has a strong inhibitory effect on AUB (Figure 1C).

#### 2.1.3. Coagulation Function Assessment

In terms of coagulation function, compared with the control group, the model group showed significant prolongation of prothrombin time (PT), activated partial thromboplastin time (APTT), and thrombin time (TT) (*p* < 0.01 or *p* < 0.05), and a significant decrease in fibrinogen (FIB) (*p* < 0.05). Compared with the model group, the DXL and BYJ group had significantly shortened PT, APTT and TT (*p* < 0.01 or *p* < 0.05), and the FIB levels were significantly increased (*p* < 0.01). In addition, APTT showed a certain trend of shortening, but there was no significant difference compared with the model group. These results indicate that the DXL and BYJ group have strong therapeutic effects on AUB rats (Figure 1D).

#### 2.1.4. Hormone Levels Assessment

Compared with the control group, the model group rats had significantly lower levels of follicle stimulating hormone (FSH, *p* < 0.01), luteinizing hormone (LH, *p* < 0.01), estrogen (E_2_, *p* < 0.05), and progesterone (Pro, *p* < 0.05). Furthermore, compared with the model group, the BYJ group had significantly increased hormone levels of FSH (*p* < 0.01), LH (*p* < 0.01), E_2_ (*p* < 0.01) and Pro (*p* < 0.05), which is consistent with the trend of the positive drug group regarding hormone levels (Figure 1E).

### 2.2. Metabolite Profile of AUB Rats and Multiple Statistic Snalysis

**Figure 2:**
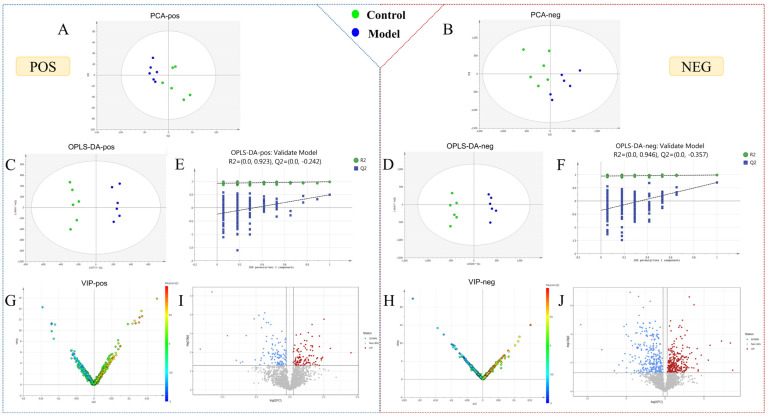
Statistical analysis of urine metabolomics. (**A**–**D**) PCA score plots and OPLS-DA score plot for the control and model groups on the 7th day. (**E**,**F**) Permutation test of OPLS-DA in positive and negative ion mode. (**G**,**H**) VIP plot of the control and model groups in positive and negative ion mode. (**I**,**J**) Volcano plot of the control and model groups in positive and negative ion mode.

We obtained the metabolomic data of urine samples using the optimal chromatography–mass spectrometry method (Figures S1 and S2), which was then imported into Progenesis QI for chromatographic peak alignment, data normalization, peak extraction, and multivariate statistical analysis. First, principal component analysis (PCA) and orthogonal partial least squares-discriminant analysis (OPLS-DA) were performed with the metabolomic data collected on day 7 of the modeling. The results showed that the overall metabolic profile of urine samples from the control and model groups displayed intragroup clustering and intergroup separation, indicating significant changes to the metabolic network of the model group (Figure 2A–D). In the OPLS-DA model, the R^2^Y (cum) and Q^2^ (cum) values of each group were greater than 0.9, indicating that the model was well built and had reliable predictability. The permutation test (*n* = 200) of model parameters R^2^ and Q^2^ confirmed that there was no overfitting phenomenon (Figure 2E,F). Specific parameters are shown in Table S1.

### 2.3. Potential Biomarker Identification of AUB Model

To identify endogenous metabolites that play a crucial role in metabolic profile changes, OPLS-DA analysis was performed. VIP plots and Volcano plots (Figure 2G–J) were further obtained to intuitively reflect the contribution of ions to the significant changes in metabolic profile. Ions located further away from the origin in the plot indicated a greater contribution to the inter-group difference and were more likely to be potential biomarkers for AUB. Endogenous ions with variable importance in projection (VIP) > 1, *t*-test of intergroup changes (*p* < 0.05) and fold change (FC) > 1.2 were selected as candidate biomarkers. The potential biomarker ions were identified or characterized by matching their accurate mass, molecular formula, and MS/MS data with databases such as HMDB, MassBank, and ChemSpider, as well as by combining chemical structure analysis and mass spectrometry fragmentation patterns.

**Figure 3:**
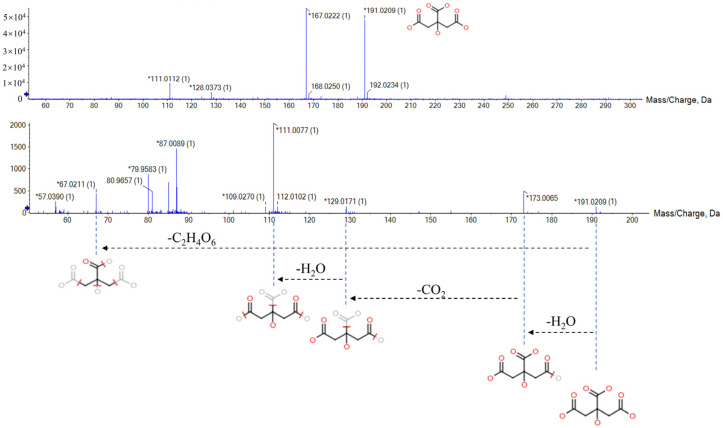
The MS/MS information and fragmentation pattern of citric acid in negative ion mode.

Citric acid can be used as an example to illustrate the identification process of biomarkers (Figure 3). In the negative ion mode, the differential ion of Rt_*m*/*z* 1.68_191.0209 having VIP and FC values of 5.6307 and 1.469, respectively, might be the dehydrogenated ion of citric acid, which has a molecular formula of C_6_H_8_O_7_. Additionally, characteristic fragment ions in the MS/MS spectrum included *m*/*z* 173 [M-H-H_2_O]^−^, *m*/*z* 129 [M-H-H_2_O-CO_2_]^−^, *m*/*z* 111 [M-H-H_2_O-CO_2_-H_2_O]^−^, and *m*/*z* 76 [M-H-C_2_H_4_O_6_]^−^. In accordance with the fragmentation pathway, we identified this ion as citric acid. Finally, we identified a total of 32 biomarkers of AUB model using the same approach (Table 1).

### 2.4. Analysis of Metabolic Pathway Associated with AUB

**Figure 4:**
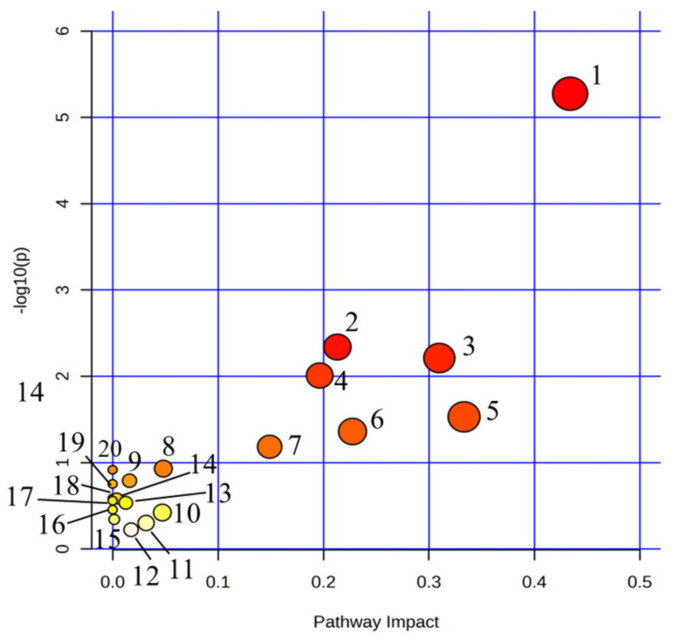
Summary of pathway analysis with MetPA on the potential biomarkers in AUB rats. 1. Arachidonic acid metabolism; 2. Steroid hormone biosynthesis; 3. Glycerophospholipid metabolism; 4. Tryptophan metabolism; 5. alpha-Linolenic acid metabolism; 6. Retinol metabolism; 7. TCA cycle; 8. Alanine, aspartate and glutamate metabolism; 9. Purine metabolism; 10. Pentose phosphate pathway; 11. Glyoxylate and dicarboxylate metabolism; 12. Tyrosine metabolism; 13. Glycerolipid metabolism; 14. Glycosylphosphatidylinositol (GPI)-anchor biosynthesis; 15. Phosphatidylinositol signaling system; 16. Ether lipid metabolism; 17. Butanoate metabolism; 18. Arginine biosynthesis; 19. Biosynthesis of unsaturated fatty acids; 20. D-Glutamine and D-glutamate metabolism.

**Figure 5:**
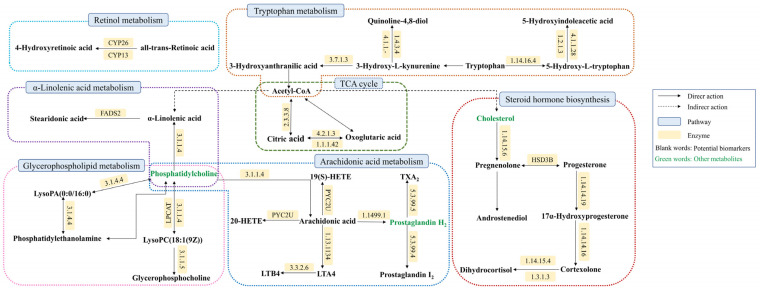
Association networks of the potential biomarkers based on the KEGG.

We imported the 32 urine biomarkers into the MetaboAnalyst 5.0 online platform to search for metabolic pathways closely related to AUB and found 20 metabolic pathways with abnormalities (Figure 4, Table S2). Among them, the impact represents the hit nodes, and the larger the impact, the more hit nodes. Taking an impact value of >0.1 as the screening criterion, 7 core metabolic pathways most closely related to urine metabolites in AUB were obtained, including arachidonic acid metabolism, steroid hormone biosynthesis, glycerophospholipid metabolism, tryptophan metabolism, α-linolenic acid metabolism, citrate cycle (TCA cycle), and retinol metabolism (Figure 5).

### 2.5. Effects of BYJ on AUB Rats Based on Urine Metabolite Profile

**Figure 6:**
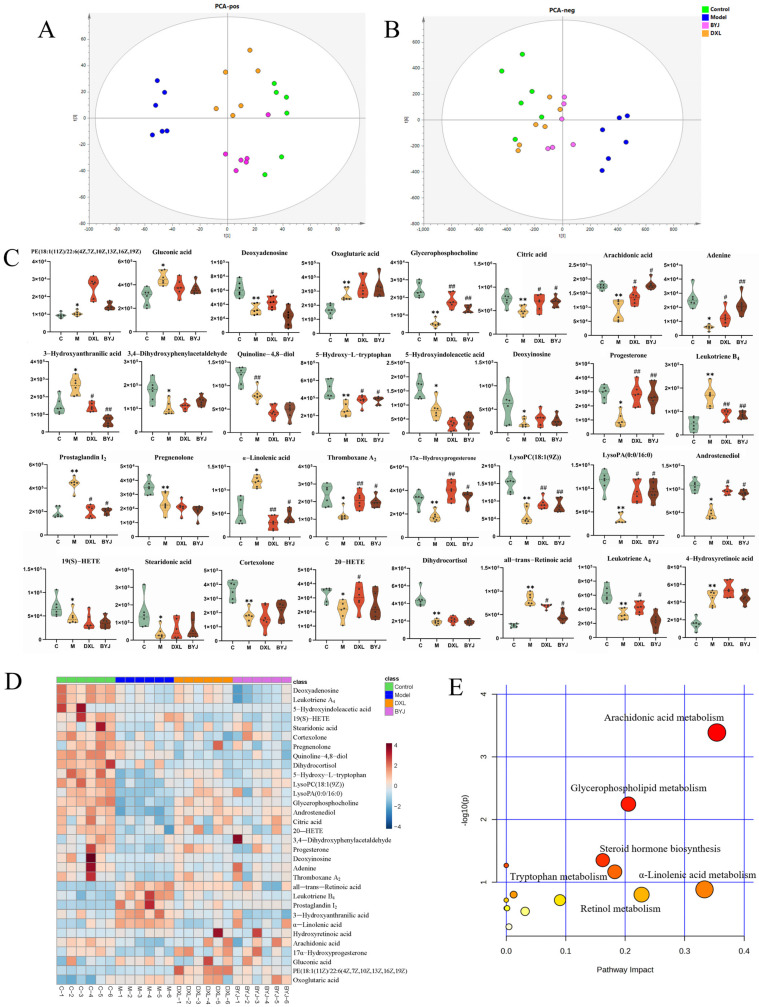
Regulation effect and related metabolic pathway analysis of BYJ on potential biomarkers in AUB rats. (**A**) PCA score plots in positive mode. (**B**) PCA score plots in negative mode. (**C**) The relative intensity of urine potential biomarkers between different groups * *p* < 0.05, ** *p* < 0.01 compared with the control group; ^#^ *p* < 0.05, ^##^ *p* < 0.01 compared with the model group. (**D**) Heatmap of the differences in the potential biomarkers between different groups. (**E**) MetPA metabolic pathway analysis of therapeutic effect of BYJ on AUB model rats.

EZinfo 3.0 was further used to perform PCA analysis (Figure 6A,B). The results showed that the model group and the control group were still significantly separated, the BYJ group and DXL group tending to be closer to the control group and significantly separated from the model group. This indicates BYJ has an effect on alleviating the pathological state of AUB.

The BYJ significantly reversed potential biomarkers of the AUB model with abnormal levels of 16/32 (Figure 6C,D), including glycerophosphocholine, citric acid, arachidonic acid, adenine, 3-Hydroxyanthranilic acid, 5-Hydroxy-L-tryptophan (5-HT), progesterone, leukotriene B_4_ (LTB_4_), prostaglandin I_2_ (PGI_2_), α-linolenic acid, thromboxane A_2_ (TXA_2_), 17α-Hydroxyprogesterone, lysoPC (18:1(9Z)), lysoPA (0:0/16:0), androstenediol, and all-trans-retinoic acid. BYJ may exert its therapeutic effect by correcting the abnormal disturbances in arachidonic acid metabolism, steroid hormone biosynthesis, glycerophospholipid metabolism, tryptophan metabolism, α-linolenic acid metabolism, retinol metabolism, and the TCA cycle by modulating the levels of the above biomarkers (Figure 6E).

### 2.6. Effective Constituents of BYJ against AUB Rats

**Figure 7:**
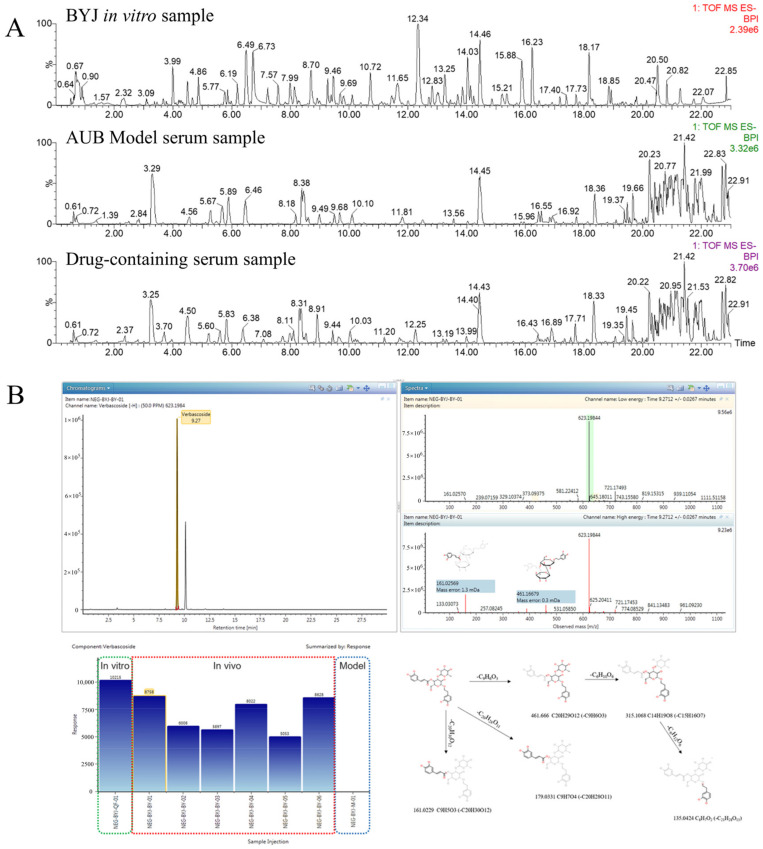
Analysis of effective components in serum affected by AUB model rats based on UNIFI software. (**A**) Chromatograms of BYJ *in vitro* sample, AUB model serum sample, and drug-containing serum sample.(**B**) The results of matching MS/MS fragment ions and trend plot of the identified absorbed compounds and cleavage law.

A high-resolution UPLC-G2-Si/MS^E^ system combined with UNIFI software was used to screen the blood components and metabolites of BYJ. The MS^E^ data of model serum samples, drug-containing serum samples, and BYJ *in vitro* samples were imported into the UNIFI software. By selecting a precise mass error range of less than 5mDa and combining MS^E^ high–low collision energy data, fragment ions, and content comparison, we characterized the peak structure and analyzed its transformation process *in vivo* (Figure 7).

Verbascoside can be used as an example to illustrate the identification process of blood components by UNIFI software (Figure S5). In negative ion mode, *m*/*z* 623.1984 [M-H]^−^ was detected in drug-containing serum at 7.29 min, with a molecular formula of C_29_H_36_O_15_.Under high-energy collision conditions, the quasi-molecular ion was further fragmented to yield the following MS/MS fragment ions: 461 Da [M-H-C_9_H_6_O_3_]^−^, 315 Da [M-H-C_9_H_6_O_3_-C_6_H_10_O_6_]^−^, and 135 Da [M-H-C_9_H_6_O_3_-C_6_H_10_O_6_-C_6_H_12_O_6_]^−^. The characteristic fragment ion at 179 Da was generated by loss of the terminal caffeic acid, while the ion at 161 Da represented caffeoylbenzyl alcohol glycoside. The mass spectrometric fragmentation pathway was consistent with the literature and reference substance, confirming the compound’s identity as verbascoside.

Finally, a total of 59 blood components were identified using the UNIFI database, including 39 prototype components and 20 metabolized components (Table S3). These metabolites were mainly produced through Phase I and Phase II metabolism reactions such as oxidation, reduction, hydrolysis, glucuronidation, and sulfation. Of the 59 identified blood components, 12 from Rehmanniae Radix and Rehmanniae Radix Praeparata, 8 from Paeoniae Radix Alba, 11 from Phellodendri Chinensis Cortex, 11 from Scutellariae Radix, 7 from Dipsaci Radix, 2 from Dioscoreae Rhizoma, and 11 from Glycyrrhizae Radlx et Rhizoma. Some of these compounds were shared by multiple herbs, such as gallic acid, which is shared by Paeoniae Radix Alba. and Dioscoreae Rhizoma, or chlorogenic acid and cryptochlorogenic acid, which are shared by Phellodendri Chinensis Cortex and Dipsaci Radix.

### 2.7. Correlation Analysis of between Biomarkers and Absorbed Components

**Figure 8:**
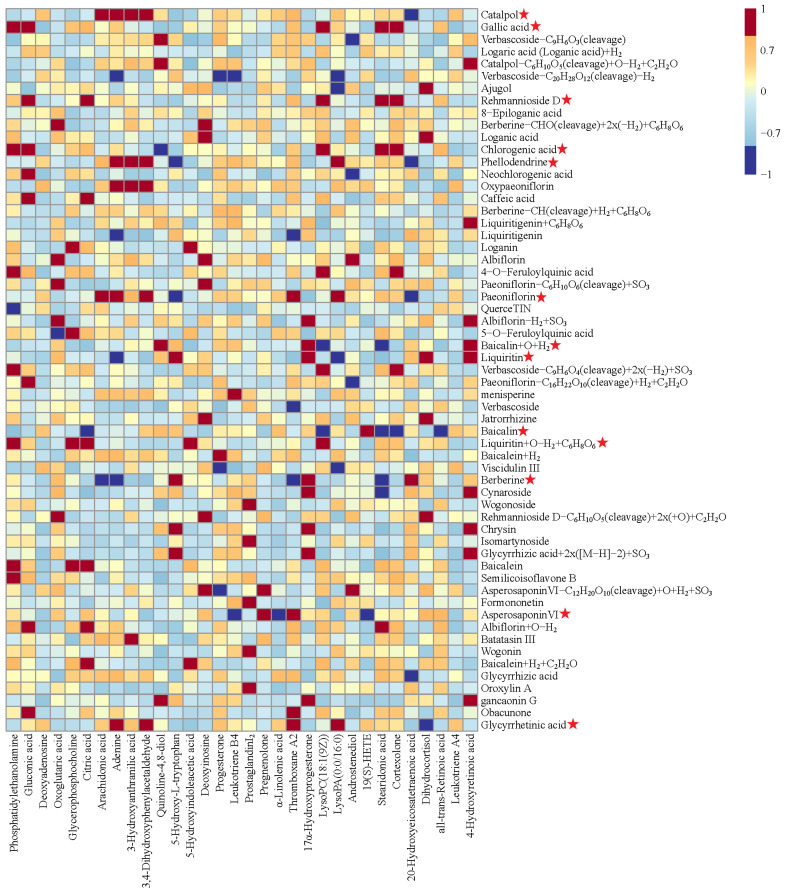
Pearson correlation analysis of blood components of BYJ and potential biomarkers in AUB rats. (“
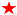
”: potential effective constituents).

The potential effective constituents of BYJ were screened by Pearson correlation analysis. Finally, 13 components were identified as correlated with the efficacy of BYJ, including catalpol, gallic acid, rehmannioside D, chlorogenic acid, phellodendrine, paeoniflorin, liquiritin, baicalin, liquiritin+O−H_2_+C_6_H_8_O_6_, berberine, asperosaponinVI, baicalin+H_2_+C_2_H_2_O, and glycyrrhetinic acid (Figure 8, Table S4).

### 2.8. Determination of Q-Markers for BYJ

**Figure 9:**
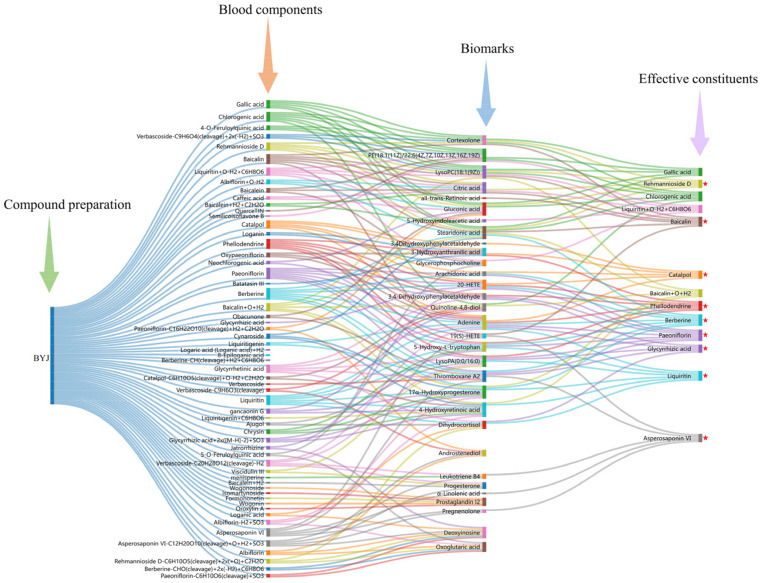
The diagram for the connection of blood components–biomarkers–potentially effective constituents. (“
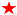
”: Q-markers).

Under the guidance of Chinmedomics, 13 active ingredients closely related to AUB were screened. Combined with the “Five Principles” of the Q-markers, catalpol, rehmannioside D, paeoniflorin, berberine, phellodendrine, baicalin, asperosaponinVI, liquiritin, and glycyrrhizic acid were identified as quality markers of BYJ (Figure 9).

## 3. Discussion

Numerous women experience physical, social, and emotional distress due to AUB. Previous studies have indicated that endometrial vascular damage and uterine tissue inflammation are the main clinical features of AUB [12]. Hormonal imbalance is also a major pathological mechanism for uterine bleeding [13,14]. Research has demonstrated that an increase in pro-angiogenic factors and a decrease in anti-angiogenic factors can impair vascular maturation, leading to more fragile blood vessels and consequently AUB [15]. Therefore, promoting endometrial and vascular repair and regulating immune responses may help to suppress the occurrence of AUB. BYJ is a classic prescription for treating AUB, with the effects of nourishing *yin*, clearing heat, and hemostasis. However, the specific mechanism action of BYJ is largely unknown, and its Q-markers are not yet clear, which has hindered the development of new drugs based on BYJ.

In this study, the AUB model was simulated by incomplete medical abortion, reflecting the characteristics of metrorrhagia in TCM syndromes. After incomplete medication abortion, the physiological function of HPOA in rats was disordered, and the gonadotropin-releasing hormone (GnRH) secreted by hypothalamus was reduced, which in turn led to the decrease of FSH and LH secreted by pituitary and the decrease of Pro and E_2_ secreted by ovary; additionally, the coagulation system of the body was activated, PT, APTT and TT were significantly prolonged, and FIB was significantly decreased. By studying the changes to pathological tissue, uterine bleeding, coagulation function, and hormone levels in rats after drug administration, it was confirmed that BYJ can obviously increase the hormone levels of FSH, LH, E_2_ and Pro, thus adjusting the physiological function of the HPOA axis, adjusting the disorder of coagulation system caused by incomplete medical abortion, obviously shortening PT, APTT and TT, and increasing FIB level, thus reducing the uterine bleeding volume in rats and showing a good hemostatic effect.

Metabolomic techniques can better reveal the pathological characteristics of diseases, help to discover the targets of drugs, and explain their overall mechanisms of action. Here, metabolomics techniques were used to identify biomarkers of AUB, and the metabolic pathways and pathogenesis of AUB, as well as the hemostatic mechanism of BYJ, were preliminarily elucidated. In this study, we identified 32 potential biomarkers of AUB; after treatment with BYJ, 16 abnormal metabolic products were significantly reversed, mainly involving lipid metabolism, amino acid metabolism, and carbohydrate metabolism.

Arachidonic acid (AA) metabolism is a key pathway in the development of AUB, and it plays an important role in various physiological and pathological processes such as coagulation balance and inflammatory response. AA is an unsaturated fatty acid that plays an important role in the structure and function of cell membranes in the human body. It can be converted into various metabolites such as prostaglandins (PGs), thromboxanes (TXs), leukotrienes (LTs), and hydroxyeicosatetraenoic acids (HETEs) through the action of cyclooxygenase and lipoxygenase [16]. TXA_2_ and PGI_2_ are vasoactive substances, which are endogenous metabolites closely related to the formation mechanism of AUB and regulate hemodynamics in the endothelium. TXA_2_ has a strong vasoconstrictive effect and promotes platelet aggregation and release [17], while PGI_2_ is synthesized by the endothelial cells of blood vessels and has vasodilatory and platelet-inhibiting effects. Under normal conditions, TXA_2_ and PGI_2_ are in a dynamic balance, jointly regulating vasoconstriction and dilation and participating in the regulation of blood clotting [18]. In patients with AUB, the release of AA and the secretion of TXA_2_ are reduced, while the content of PGI_2_ increases significantly, resulting in an imbalance of the TXA_2_/PGI_2_ ratio, leading to vasodilation, local blood flow increase, and increased local bleeding. On the other hand, AA is converted into leukotrienes (including LTA_4_ and LTB_4_) under the action of 5-lipoxygenase. Leukotrienes are strong inflammatory factors that can participate in smooth muscle contraction and mediate inflammatory reactions. In addition, AA metabolized through the CYP pathway produces hydroxyeicosatetraenoic acids (HETEs), including 19-HETE and 20-HETE. These C20 fatty acids have important regulatory effects on blood rheology, vascular elasticity, leukocyte function, and platelet activation. They can promote vasoconstriction and control or alter the vascular delivery system [19,20]. In our study, it was found that the content of PGI_2_ and LTB_4_ in the AUB model group of rats was significantly increased, while the content of AA, TXA_2_, LTA_4_, 19-HETE, and 20-HETE was significantly decreased. This indicates that AA metabolism is disrupted, leading to an increase in PG synthesis and a decrease in TX and HETE synthesis, which inhibit platelet aggregation, uterine smooth muscle contraction, and vasoconstriction, resulting in increased bleeding and prolonged bleeding time. On the other hand, the increase in LTB_4_ leads to increased inflammation. Compared with the model group, the BYJ-dosed group could significantly modulate the disordered status of arachidonic acid, TXA_2_, PGI_2_, LTB_4_ and 20-HETE in rats with AUB, regulate platelet aggregation and release, promote the contraction of blood vessels and uterine smooth muscle, and thus exert the effect of blood clotting.

In our study, we found that the underlying mechanism of AUB is closely related to the synthesis of steroid hormones, including the metabolism products of common steroids such as pregnenolone, progesterone, 17α-hydroxyprogesterone, and dihydrocortisol. Steroids are a class of biologically active substances derived from cholesterol, which is catalyzed by cholesterol side-chain cleavage enzyme (CYP11A1) to produce pregnenolone, a precursor to most steroid hormones [21]. Pregnenolone is then converted to progesterone by the action of 3beta-hydroxy-Delta5-steroid dehydrogenase, which has an inhibitory effect on uterine smooth muscle contraction [22]. Progesterone is further converted to 17α-hydroxyprogesterone by 17α-monooxygenase. In the rat model of AUB, the normal physiological function of the HPOA is disrupted after incomplete abortion, leading to reduced secretion of uterine endometrial steroid hormones and lysosomal enzymes, and decreased uterine smooth muscle contraction, resulting in uterine bleeding. Our study found that pregnenolone, progesterone, 17α-hydroxyprogesterone, cortisol, dihydrocortisol, and testosterone were disturbed, and the concentrations of these markers were significantly lower in the model group compared to the control group. BYJ might therefore increase the secretion of uterine endometrial steroid hormones and lysosomal enzymes and promote uterine smooth muscle contraction, which is beneficial for hemostasis and repair of the endometrium.

Glycerophospholipid metabolism mainly involves metabolites such as lysophosphatidic acid (LysoPA), lysophosphatidylcholine (LysoPC), lysophosphatidylethanolamine (LysoPE), and lysophosphatidylinositol (LysoPI). These lysophospholipids are widely present in cell membranes of organisms and mainly participate in the synthesis of various phospholipids. Among them, lysophosphatidic acid (LysoPA), which is a cell-signaling molecule closely related to the occurrence and development of vascular endothelial smooth muscle-related diseases, is the simplest structure of phospholipids. LysoPA can cause a series of vascular reactions, including platelet aggregation, smooth muscle contraction, and stimulation of smooth muscle cell proliferation [23]. Phosphatidylcholine is a phospholipid mediator with multiple biological activities and which performs important biological functions in vascular smooth muscle diseases. Phosphatidylcholine can be hydrolyzed into lysophosphatidylcholine by phospholipase A, thereby promoting platelet aggregation and stimulating smooth muscle cell proliferation [24,25]. In this study, these markers were disrupted in the model group, consistent with literature reports, and can be used as diagnostic markers for AUB. Moreover, BYJ significantly reversed the disorder of LysoPC (18:1(9Z)), Glycerophosphocholine, and LysoPA (0:0/16:0) in the rat model of AUB, ultimately achieving a therapeutic effect in stopping bleeding.

α-Linolenic acid is an essential fatty acid in the human body with important physiological activities. It competes with cyclooxygenase and lipoxygenase in cell membrane phospholipids, inhibits the production of TXA_2_, and generates TXA_3_ and PGI, thus inhibiting platelet aggregation and dilating blood vessels [26]. In this study, the content of α-linolenic acid in the model group increased significantly, and compared with the model group, BYJ can significantly reduce the content of α-linolenic acid and restore the production of TXA_2_, thus regulating the disorder of arachidonic acid, TXA_2_, PGI_2_, LTB_4_ and 20-HETE in rats with AUB and regulating platelet aggregation and release.

In addition, tryptophan is an essential amino acid required for cell proliferation and differentiation in animals. 5-Hydroxy-L-tryptophan (5-HT) is an important messenger and neuromodulator in the body that promotes platelet aggregation, smooth muscle cell proliferation, and enhances the vasoconstrictive effect of vasoconstrictors, thereby playing a role in hemostasis. 5-Hydroxyindoleacetic acid is a metabolite of the 5-HT pathway, and studies have shown that indole and its derivatives are cytotoxic metabolites that can cause vascular dysfunction [27,28]. In our study, the content of 5-HT and 5-Hydroxyindoleacetic acid in the model group rats was significantly reduced. Compared with the model group, the BYJ-dosed group significantly adjusted the contents of 5-HT and 5- hydroxyindoleacetic acid, which promotes the contraction of blood vessels and uterine smooth muscle and plays a role in coagulation.

A total of 59 compounds that directly act on the body for the treatment of AUB were identified in BYJ by using the serum pharmacochemistry technology of TCM. Moreover, 13 compounds highly correlated with potential biomarkers of AUB rats were selected by analyzing the correlation between blood components and potential biomarkers. These 13 compounds were catalpol, gallic acid, rehmannioside D, chlorogenic acid, phellodendrine, paeoniflorin, liquiritin, Baicalin, liquiritin+O−H_2_+C_6_H_8_O_6_, berberine, asperosaponinVI, baicalin+H_2_+C_2_H_2_O, and glycyrrhetinic acid. However, gallic acid and chlorogenic acid are not unique components of a single herb and do not meet the “Five Principles” of quality markers. Liquiritin+O−H_2_+C_6_H_8_O_6_ is a metabolite of liquiritin, glycyrrhetinic acid is a metabolite of glycyrrhizic acid, and baicalin+H_2_+C_2_H_2_O is a metabolite of baicalin. Therefore, based on the “Five Principles” of Q-markers [29], 9 of these compounds were ultimately determined to be quality markers for the treatment of AUB with BYJ, namely catalpol, rehmannioside D, phellodendrine, paeoniflorin, liquiritin, baicalin, berberine, asperosaponinVI, and glycyrrhizic acid.

Catalpol is a highly promising substance for regulating the treatment of vascular inflammatory diseases. It has strong anti-inflammatory and hemostatic functions, promotes angiogenesis, improves barrier permeability, and repairs damage to vascular endothelial cells (Zhang et al., 2020; Zhang et al., 2022). Rehmannioside D has the effect of nourishing yin and replenishing blood. Paeoniflorin has immunomodulatory and anti-inflammatory effects and can inhibit PDGF-BB-induced vascular smooth muscle cell (VSMC) proliferation through the ROS-mediated ERK1/2 and p38 signaling pathways [30]. Berberine hydrochloride reduces lipopolysaccharide-induced endometritis in mice by suppressing activation of the NF-κB signal pathway [31]. Berberine hydrochloride also has the potential to treat various chronic inflammatory diseases by activating NF-κB in mononuclear cells to inhibit the inflammatory response [32]. Baicalin protects against endometritis by inhibiting the NF-κB and JNK signaling pathways and pro-inflammatory cytokines, reducing inflammatory cell infiltration, congestion, bleeding, and epithelial cell shedding. It also protects against decidual cell damage in LPS-induced miscarriage mice [33,34]. Saponin components can inhibit vascular smooth muscle proliferation and platelet aggregation. Asperosaponin VI has a progesterone-like effect and promotes decidualization by regulating the expression of key targets (JUN, CASP3, STAT3, SRC, and PTGS2) in decidual cells through the activation of PR expression and the Notch signaling pathway, thereby treating recurrent spontaneous abortion [35,36]. Liquiritin significantly activates the Keap1/Nrf2/HO-1 signaling pathway, thereby inhibiting cell oxidative stress and inflammatory reactions and effectively protecting the endometrium [37]. Glycyrrhizic acid significantly affects rat uterine tissue contraction by regulating the biosynthesis of steroids and reducing uterine smooth muscle contraction [38].

## 4. Materials and Methods

### 4.1. Reagents and Materials

HPLC-grade methanol and acetonitrile were purchased from Thermo Fisher Scientific (Waltham, MA, USA), and formic acid and leucine enkephalin were purchased from Sigma-Aldrich (Shanghai, China). APCI Positive/Negative Calibration (AB SCIEX, Framingham, MA, USA) and distilled water were obtained from Watsons (Guangzhou, China). Other analytical grade reagents were purchased from Beijing Institute of Chemical Reagents Co., Ltd.). ELISA kits including follicle-stimulating hormone (FSH, Lot number: H101-1-2), luteinizing hormone (LH, Lot number: H206-1-2), estrogen (E_2_, Lot number: H102-1-2), and progesterone (Pro, Lot number: H089-1-2) were provided by Nanjing Jiancheng Bioengineering Research Institute.

Mifepristone and misoprostol were obtained from Zizhu Pharmaceutical Co. (Peking, China). The Duanxueliu pills used were produced by Anqing Huiyinbi Pharmaceutical Co., Ltd., (Anqing, China). The following were provided by Shenwei Pharmaceutical Co. Ltd. (Shijiazhuang, China): Rehmanniae Radix (Lot number: HNJZ20191201); Rehmanniae Radix Praeparata (Lot number: HNJZ20191203); Paeoniae Radix Alba (Lot number: SCZ20201013); Phellodendri Chinensis Cortex (Lot number: HBL2011091); Scutellariae Radix (Lot number: SXSL20191101); Dipsaci Radix (Lot number: SCYY2011091); Dioscoreae Rhizoma (Lot number: 20201101); and Glycyrrhizae Radlx et Rhizoma. (Lot number: NMHJQ20191102); these were authenticated by Professor Xijun Wang in the Department of Pharmacognosy, Heilongjiang University of Chinese Medicine.

### 4.2. Preparation of Medicinal Drugs

The decoction method recorded in *Jingyue Quanshu* was used to prepare BYJ samples. According to metrological verification, the modern formulation of BYJ was as follows: Rehmanniae Radix: 7.46 g; Rehmanniae Radix Praeparata: 7.46 g; Paeoniae Radix Alba: 7.46 g; Phellodendri Chinensis Cortex: 5.60 g; Scutellariae Radix: 5.60 g; Dipsaci Radix: 5.60 g; Dioscoreae Rhizoma: 5.60 g; and Glycyrrhizae Radlx et Rhizoma: 3.73 g. The mixture of above herbs were combined with 400 mL of water and boiled to 140 mL. We collected the filtrate through 140-mesh gauze and dried it into lyophilized powder. The average powder yield was 29.57% (*n* = 15). The powder was stored at room temperature in a desiccator, and an appropriate amount of freeze-dried powder was dissolved in distilled water (0.6 g/mL) for later use.

The content of paeoniflorin, berberine, baicalin, asperosaponinVI, and glycyrrhizic acid and their fingerprints were used as indicators of the quality of the above extracts. The RSD values of the above components in five batches of freeze-dried powder were 1.23%, 1.80%, 1.30%, 1.26%, and 1.66% respectively (Figure S6, Table S5). The similarity of the fingerprints of 15 batches of BYJ is greater than 0.9 (Figure S7, Table S6). This indicates that the prepared samples are stable and controllable.

### 4.3. Animal Handling

Sprague–Dawley (SD) female rats (body weight: 250 ± 10 g) were purchased from the Experimental Animal Center of Heilongjiang University of Traditional Chinese Medicine (Harbin, China). The SD rats were raised in an environment at 23 ± 2 °C with 50 ± 5% relative humidity and a 12 h/12 h light/dark cycle with free access to food and water. All the rats were acclimated for seven days prior to experimentation.

**Figure 10:**
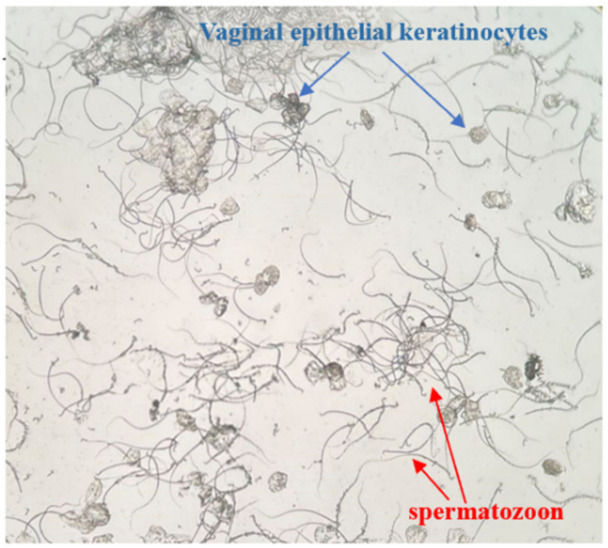
Female rat vagina image (×100).

Female rats in proestrus or estrus were mated with male rats in a separate cage at a ratio of 2:1 overnight, and the vaginal secretions of females were collected for vaginal smear examination the next morning [39,40,41]. As soon as sperm and keratoepithelial cells were found, it was considered the first day of pregnancy (Figure 10). Pregnant rats were randomly divided into three groups (*n* = 6): the AUB model group, positive control group with Duanxueliu solution (DXL), and treatment group (BYJ). On day 7 of pregnancy, each rat was orally administered mifepristone (8.5 mg/kg, 8:00 am) and misoprostol (0.1 mg/kg, 6:00 pm) to establish an AUB model of early-pregnancy incomplete medical abortion. An additional six non-pregnant normal rats were used as the control group.

Concurrently, each group of rats received the corresponding drugs by gavage on the first day after modeling. For a period of 7 days, the control and model groups were given equal volumes of normal saline, the positive control group received oral administration of DXL at a dose of 0.91 mg/kg/d, and the treatment group was administered BYJ at a dose of 13.97 mg/kg/d. The experimental protocol was reviewed and approved by the Ethics Committee of Heilongjiang University of Chinese Medicine.

### 4.4. Pharmacodynamic Evaluation of BYJ on AUB Rats

#### 4.4.1. Morphological and Pathological Analysis

The fresh uterine tissue was fixed in 4% paraformaldehyde for 48 h after photographing its complete morphology, and hematoxylin and eosin (HE) staining was performed to observe pathological changes in the uterine endometrium under a microscope.

#### 4.4.2. Uterine Bleeding Volume Evaluation

After modeling, a quantitative cotton ball (60 ± 5 mg) was placed in the vagina of each rat to absorb uterine bleeding, and the cotton ball was wrapped with plastic film to prevent blood leakage. The cotton ball was removed from the vagina and placed in a sealed EP tube for refrigeration at 8:00 a.m. and 6:00 p.m. of each day. The amount of vaginal bleeding was observed and recorded, and a new cotton ball was replaced in the vagina; this procedure continued until the 14th day.

On the 7th day after administration, 0.02 mL of blood was collected from each rat’s tail vein and added to 4 mL of 5% NaOH solution (V_1_), mixed and diluted for use. Each collected vaginal cotton ball from the rats was placed in a beaker and washed with an appropriate volume of 5% NaOH solution according to the amount of uterine bleeding in the rats. The above procedure was repeated 1–2 times until the cotton ball was washed to a white color, and the total volume of 5% NaOH solution (V_2_) used was recorded. A total of 5 mL of the extracted solution from the washed cotton ball and 4 mL of 5% NaOH solution containing rat tail vein blood were taken and compared to the blank control with 5% NaOH solution. The absorbance value (A) was detected using a microplate reader (546 nm wavelength) [41]. The formula for the volume of uterine bleeding was as follows:

Volume (mL) = tail vein blood (0.02 mL) × V_2_ × uterine blood extract (A_1_)/tail vein blood (A_2_) × V_1_

#### 4.4.3. Coagulation Function Test

This study used biochemical indexes of prothrombin time (PT), activated partial thromboplastin time (APTT), thrombin time (TT), and fibrinogen (FIB) to evaluate the coagulation function of AUB rats. On the 7th day after administration, the rats were terminated, and blood was collected. After standing at room temperature for 1.5 h, the serum was obtained by centrifugation at 3000 rpm for 10 min, and the above indexes were measured by fully automated hemagglutination analyzer.

#### 4.4.4. Hormone Levels Assessment

The blood was collected and placed at room temperature for 1.5 h and centrifuged at 3000 rpm for 10 min to obtain the serum, and FSH, LH, E_2_, and Pro were determined according to the operating instructions of the corresponding kits.

### 4.5. Metabolomics Analysis

#### 4.5.1. Sample Collection and Preparation

Urine samples were collected from rats placed in metabolic cages for 12 h overnight and the urine samples were centrifuged for 10 min (4 °C, 13,000 rpm). The supernatant was diluted 2 times with HPLC-grade ultrapure water, vortexed for 1 min, then filtered through a 0.22 μm membrane prior to AB SCIEX Triple TOFTM 5600+ analysis.

#### 4.5.2. Metabolomics Analysis Conditions

The Waters ACQUITY UPLCTM system was used for chromatographic separation, and a HSS T3 (2.1 mm × 100 mm, 1.8 μm) chromatography column was maintained at a temperature of 40 °C and a flow rate of 0.4 mL/min. The injection sample volume was 4 μL. The mobile phase consisted of 0.1% formic acid in acetonitrile (solvent A) and 0.1% formic acid in water (solvent B), and the gradient eluting conditions were stated as follows: 0–2 min, 1–11% A; 2–2.3 min, 11–21% A; 2.3–5 min, 21–40% A; 5–8 min, 40–100% A.

The mass spectrometry analysis was performed using an AB SCIEX Triple TOFTM 5600+ analysis system with electrospray ionization (ESI). The ion spray voltage was 5500 V in positive ion mode and 4000 V in negative ion mode. The nebulizer gas was set to 55 psi, and the desolvation gas was set to 55 psi. The ion source temperature was 600 °C. The MS scan declustering potential (DP) was 100 V/−100 V, and the collision energy (CE) was 10 Ev with an accumulation time of 100 ms. The IDA scan CE was set at 35 V/−35 V with a collision energy spread (CES) of 15 and an accumulation time of 50 ms. The mass scan range was set at 50–1200 Da. The automatic calibration delivery system (CDS) used APCI and an external standard calibration method for automatic adjustment and calibration of MS and MS/MS.

### 4.6. Serum Pharmacochemistry Analysis

#### 4.6.1. Serum Sample Preparation

The serum pharmacochemistry analysis was performed on samples from the model and BYJ groups. Phosphoric acid of 40 μL was added to 2 mL of serum, and the mixture was sonicated for 1 min and vortexed for 30 s. The sample was loaded onto the activated HLB solid-phase extraction (SPE) column, washed with 2 mL water, and eluted with 2 mL methanol. The methanol eluate was collected and dried with nitrogen at 37 °C, and the residue was dissolved in 60% methanol (200 μL) by sonication. After centrifugation for 10 min (4 °C, 13,000 rpm), the supernatant was collected for UPLC-MS analysis.

#### 4.6.2. Constituents Analysis Conditions

Chromatographic analysis was conducted on Waters Acquity^TM^ UPLC System, and a Waters Acquity^TM^ UPLC HSS T3 (2.1 mm × 100 mm, 1.8 μm) (Waters Corp., Milford, MA, USA) chromatography column was used at a temperature of 50 °C and a flow rate of 0.4 mL/min. The injection sample volume was 5 μL. The mobile phase consisted of 0.1% formic acid in acetonitrile (solvent A) and 0.1% formic acid in water (solvent B), and the gradient eluting conditions were as follows: 0–2 min, 5–13% A; 2–11 min, 13–25% A; 11–18 min, 25–45% A; 18–20 min, 45–70% A;20–23 min, 70–100% A.

The Waters SynaptTM G2-Si MS^E^ mass spectrometry system (Waters Corporation, Milford, MA, USA) was used to collect data in both positive and negative ion modes (ESI+, ESI−). The optimized conditions were as follows: ion source temperature 110 °C, desolvation gas temperature 350 °C, cone gas flow rate 50 L/h. In positive ion detection mode, the capillary voltage was 2.8 kV, the sample cone voltage was 20 V, the extraction cone voltage was 4.0 V, and the desolvation gas flow rate 800 L/h. In the negative ion detection mode, the capillary voltage was 2.2 kV, the sample cone voltage was 40 V, the extraction cone voltage was 4.0 V, and the desolvation gas flow was 600 L/h. Leucine enkephalin solution was used as the mass lock solution. The data acquisition interval was 0.2 s/scan, and the mass scanning range was 50–1200 Da.

#### 4.6.3. Constituent Analysis Method *In Vivo*

High-resolution UPLC-Q-TOF-MS^E^ combined with UNIFI software (Waters, the US) was used to analyze the prototypical components and metabolites of the blood. The MS^E^ data of the model serum samples, drug-containing serum samples, and BYJ *in vitro* samples were imported into UNIFI software, and 3D data-processing mode was adopted to reduce false positives and effectively match high and low energy spectra. The secondary fragments and cleavage pathways of the blood components were analyzed by using the MassFragment^TM^ module for structural confirmation.

### 4.7. Data Processing and Multivariate Statistical Analysis

The raw data were imported into Waters Progenesis QI software for peak alignment, matching, extraction, and normalization, resulting in a data matrix of ion retention time–charge ratio–peak intensity. Normalized data were imported into SIMCA 13.0 (Umetrics AB, Umea, Sweden) for multivariate statistical analysis. Principal component analysis (PCA) was used to reveal the overall differences between the groups, and orthogonal partial least squares–discriminant analysis (OPLS-DA) was used to further distinguish the characteristic variables among the groups and validate the model. Potential biomarker sets were selected based on variable importance in projection (VIP) > 1, *t*-test of intergroup changes (*p* < 0.05), and fold change (FC) > 1.2.

### 4.8. Biomarker Identification and Metabolic Pathway Analysis of AUB Model

The retention time-*m*/*z* data of potential biomarkers were matched with the compound identifications table obtained from the Progenesis QI software to obtain possible compound molecular formulas within the measurement error range. The chemical structures of biomarkers were determined by searching for their molecular formulas and molecular weights in metabolomics databases such as HMDB and KEGG, combined with MS/MS data information. Furthermore, advanced pathway analysis programs in the MetaboAnalyst platform were used to conduct pathway analysis of the identified potential biomarkers of AUB model.

### 4.9. Correlation Analysis between Biomarkers and Absorbed Constituents

The Pearson correlation analysis platform was used to calculate the correlation coefficient between *in vivo* active ingredients and biomarkers of the AUB model. The larger the value of correlation coefficient, the stronger the correlation between the two variables. In this experiment, the correlation coefficient of r = 0.7 was used as the critical value to screen highly correlated ingredients; components with 5 or more highly correlated numbers of biomarkers were considered the potential pharmacodynamic material basis of BYJ for the treatment of AUB in rats.

## 5. Conclusions

In summary, the present study adopts the Chinmedomics strategy to evaluate the therapeutic effect of BYJ on AUB in rats. Based on the effective treatment of BYJ on the AUB rat model, correlation analysis was performed between the absorbed components and the biological markers of the model, and the pharmacological material basis and mechanism of BYJ in treating AUB were clarified for the first time. The study confirms that BYJ can improve coagulation function and sex hormone levels in abnormal uterine bleeding and regulate metabolic profiles, biomarkers, and pathways. It mainly regulates arachidonic acid metabolism, steroid hormone biosynthesis, glycerol phospholipid metabolism, and tryptophan metabolism through catalpol, gallic acid, rehmannioside D, chlorogenic acid, phellodendrine, paeoniflorin, liquiritin, Baicalin, liquiritin+O−H_2_+C_6_H_8_O_6_, berberine, asperosaponinVI, baicalin+H_2_+C_2_H_2_O, and glycyrrhetinic acid. Expression of the biomarkers arachidonic acid, progesterone, lysophospholipid(0:0/16:0), and L-tryptophan were reduced to promote platelet aggregation and release and to enhance contraction of vessels and uterine smooth muscle. Moreover, using the combined “Five Principles” of Q-markers, nine components (namely catalpol, rehmannioside D, phellodendrine, paeoniflorin, liquiritin, baicalin, berberine, asperosaponin VI, and glycyrrhetinic acid) were selected as the Q-markers of BYJ for the treatment of AUB, providing a theoretical basis for the quality control of BYJ in the future. This study is helpful to further improve the internal relationship between classical prescription, pharmacodynamic material basis, and quality markers.

## Figures and Tables

**Table 1:** Specific information table of potential urine biomarkers in AUB model.

	R*_t_* (Min)	*m*/*z*	Mass Error (ppm)	Adducts	Formula	HMDB	VIP	Postulated Identity	Trend in Model
1	0.58	790.5334	−5.97	M + H	C_45_H_76_NO_8_P	HMDB0009045	3.1519	PE(18:1(11Z)/22:6(4Z,7Z,10Z,13Z,16Z,19Z)	↑
2	0.67	195.0520	4.95	M − H	C_6_H_12_O_7_	HMDB0000625	3.5397	Gluconic acid	↑
3	0.85	274.0918	2.77	M + Na	C_10_H_13_N_5_O_3_	HMDB0000101	3.7325	Deoxyadenosine	↑
4	1.12	145.0152	6.35	M − H	C_5_H_6_O_5_	HMDB0000208	3.3772	Oxoglutaric acid	↑
5	1.55	256.0944	−4.38	M − H	C_8_H_20_NO_6_P	HMDB0000086	1.6832	Glycerophosphocholine	↓
6	1.68	191.0209	6.04	M − H	C_6_H_8_O_7_	HMDB0000094	5.6307	Citric acid	↓
7	1.90	305.2442	−6.88	M + H	C_20_H_32_O_2_	HMDB0001043	1.5630	Arachidonic acid	↓
8	2.80	180.0532	3.71	M + FA − H	C_5_H_5_N_5_	HMDB0000034	1.5734	Adenine	↓
9	3.39	154.0492	−4.30	M + H	C_7_H_7_NO_3_	HMDB0001476	4.7569	3-Hydroxyanthranilic acid	↓
10	4.01	151.0409	5.47	M − H	C_8_H_8_O_3_	HMDB0003791	2.0758	3,4-Dihydroxyphenylacetaldehyde	↓
11	4.49	162.0542	−4.58	M + H	C_9_H_7_NO_2_	HMDB0060289	3.7844	Quinoline-4,8-diol	↑
12	4.69	221.0914	−2.95	M + H	C_11_H_12_N_2_O_3_	HMDB0000472	2.4592	5-Hydroxy-L-tryptophan	↓
13	5.21	190.0514	2.11	M − H	C_10_H_9_NO_3_	HMDB0000763	1.0410	5-Hydroxyindoleacetic acid	↓
14	5.39	297.0839	−0.45	M + FA − H	C_10_H_12_N_4_O_4_	HMDB0000071	2.4675	Deoxyinosine	↓
15	5.74	315.2281	0.13	M + H	C_21_H_30_O_2_	HMDB0001830	1.7058	Progesterone	↓
16	6.14	381.2288	1.63	M + FA − H	C_20_H_32_O_4_	HMDB0001085	1.0337	Leukotriene B_4_	↑
17	6.59	397.2236	−1.17	M + FA − H	C_20_H_32_O_5_	HMDB0001335	3.9050	Prostaglandin I_2_	↑
18	6.87	317.2474	−0.21	M + H	C_21_H_32_O_2_	HMDB0000253	1.1464	Pregnenolone	↓
19	7.14	323.2232	1.60	M + FA − H	C_18_H_30_O_2_	HMDB0001388	3.4682	α-Linolenic acid	↑
20	7.35	351.2181	1.19	M − H	C_20_H_32_O_5_	HMDB0001452	2.1443	Thromboxane A_2_	↓
21	7.44	331.2269	2.31	M + H	C_21_H_30_O_3_	HMDB0000374	1.8401	17α-Hydroxyprogesterone	↓
22	7.54	522.3535	−3.64	M + H	C_26_H_52_NO_7_P	HMDB0002815	2.0518	LysoPC(18:1(9Z))	↓
23	7.58	819.4761	1.03	M + FA − H	C_19_H_39_O_7_P	HMDB0007849	3.15190	LysoPA(0:0/16:0)	↓
24	7.78	335.2235	2.33	M + FA − H	C_19_H_30_O_2_	HMDB0003818	2.2561	Androstenediol	↓
25	7.80	321.2423	−0.49	M + H	C_20_H_32_O_3_	HMDB0011136	1.9988	19(S)-HETE	↓
26	7.81	299.2002	7.27	M + Na	C_18_H_28_O_2_	HMDB0006547	3.5319	Stearidonic acid	↓
27	7.83	345.2079	2.19	M − H	C_21_H_30_O_4_	HMDB0000015	3.5775	Cortexolone	↓
28	7.92	365.2335	0.33	M + FA − H	C_20_H_32_O_3_	HMDB0005998	1.0539	20-HETE	↓
29	7.97	363.2180	0.91	M − H	C_21_H_32_O_5_	HMDB0003259	1.0120	Dihydrocortisol	↓
30	8.12	301.2164	0.79	M + H	C_20_H_28_O_2_	HMDB0001852	3.0980	all-trans-Retinoic acid	↑
31	8.44	317.2127	1.38	M − H	C_20_H_30_O_3_	HMDB0001337	1.4084	Leukotriene A_4_	↓
32	8.45	315.1972	1.93	M − H	C_20_H_28_O_3_	HMDB0006254	1.3495	4-Hydroxyretinoic acid	↑

## Data Availability

Data is contained within the article and Supplementary Materials.

## Supplementary Materials

The following supporting information can be downloaded at: https://www.mdpi.com/article/10.3390/ph16050719/s1, Figure S1. Urine BPC chromatograms of the AUB model rats treated by BYJ and DXL under positive ion mode; Figure S2. Urine BPC chromatograms of the AUB model rats treated by BYJ and DXL under negative ion mode; Figure S3. Chromatograms in se-rum pharmacochemistry analysis under positive ion mode. Figure S4. Chromatograms in serum pharmacochemistry analysis under negative ion mode. Figure S5. Correlation mass spectrum and cleavage pathway of verbascoside; Figure S6. Content determination of BYJ; Figure S7. Fingerprint similarity of 15 batches of BYJ. Table S1. Model parameter of OPLS-DA; Table S2. Results of metabolic pathway analysis of AUB-related markers based on MetPA; Table S3. Identification of the components in serum after oral administration of BYJ solution; Table S4. Correlation analysis results of effective components and biomarkers; Table S5. Content determination results in five batches of BYJ; Table S6. Fingerprint similarity of 15 batches of BYJ.

## Abbreviations

5-HT	5-Hydroxy-L-tryptophan
AUB	abnormal uterine bleeding
APTT	activated partial thromboplastin time
BYJ	Baoyin Jian
E_2_	estrogen
FIB	fibrinogen
FSH	follicle stimulating hormone
HETEs	hydroxyeicosatetraenoic acids
HPOA	hypothalamic-pituitary-ovary axis
LH	luteinizing hormone
LTB_4_	leukotriene B_4_
LTs	leukotrienes
LysoPA	lysophosphatidic acid
LysoPC	lysophosphatidylcholine
LysoPE	lysophosphatidylethanolamine
LysoPI	lysophosphatidylinositol
PT	prothrombin time
Pro	progesterone
PGI_2_	prostaglandinI_2_
PGs	prostaglandins
TT	thrombin time
TXA_2_	thromboxane A_2_
TXs	thromboxanes
