# Supplementary material for: Parsing the Q-Markers of Baoyin Jian to Treat Abnormal Uterine Bleeding by High-Throughput Chinmedomics Strategy
Source: Pharmaceuticals (Basel). 2023 May 9;16(5):719. doi: 10.3390/ph16050719 (PMC10221270; doi:10.3390/ph16050719)
Supplement: Supplementary file 1 [file pharmaceuticals-16-00719-s001.zip › pharmaceuticals-2347723-supplementary.pdf]

Supplementary Material

Supplementary Figures

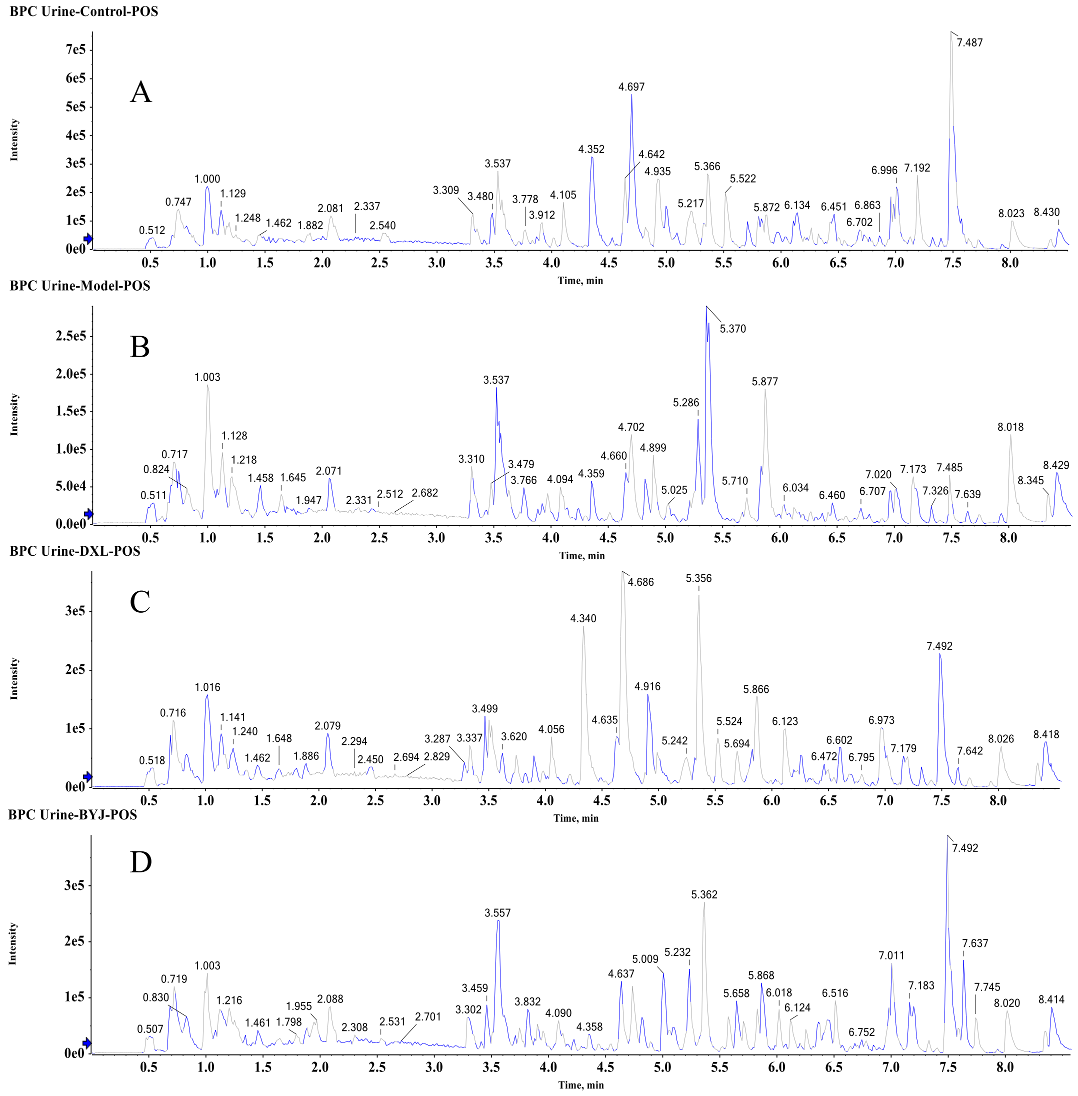

Figure S1. Urine BPC chromatograms of the AUB model rats treated by BYJ and DXL under positive ion mode. (A) Control group; (B) AUB model group; (C) DXL group; (D) BYJ group

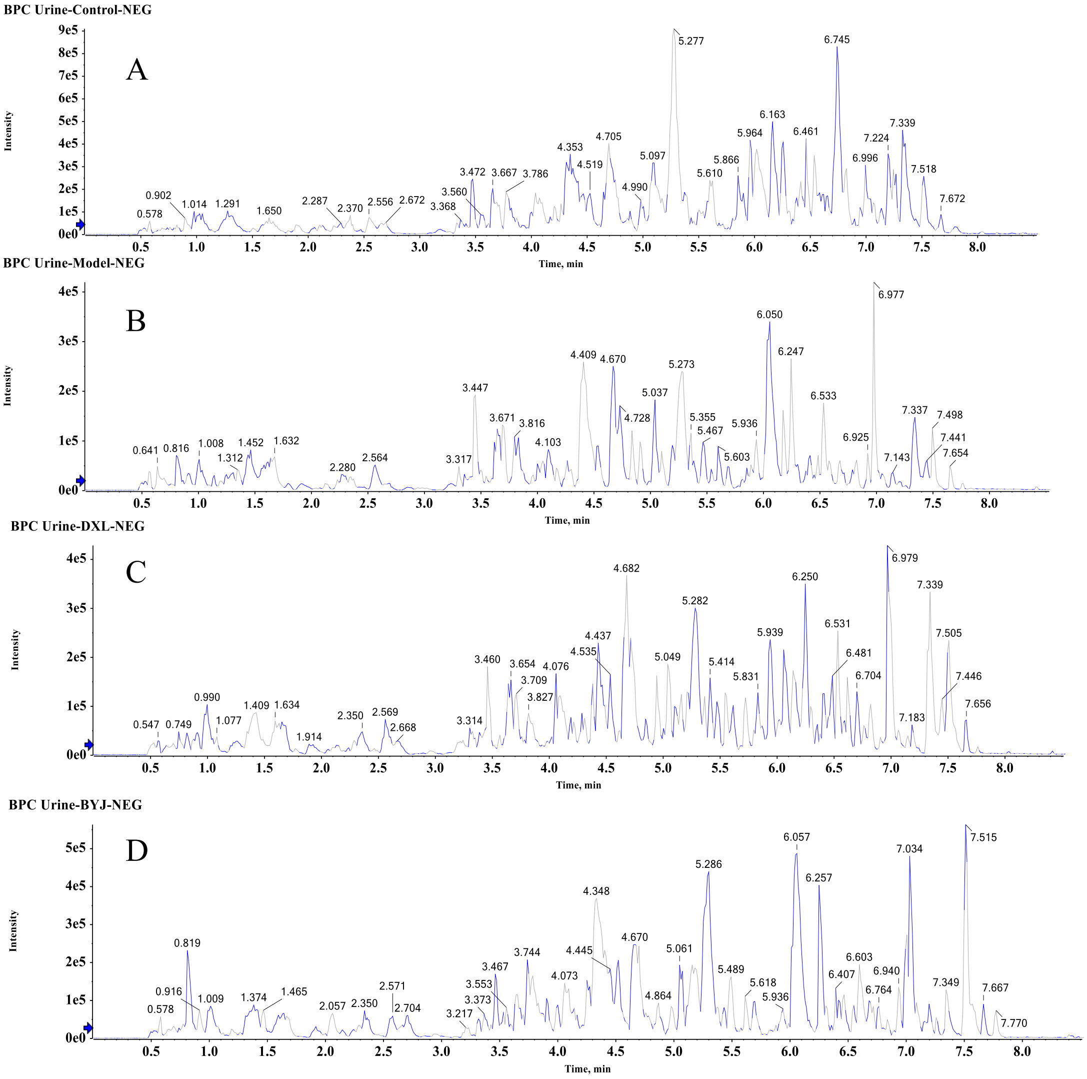

Figure S2. Urine BPC chromatograms of the AUB model rats treated by BYJ and DXL under negative ion mode. (A) Control group; (B) AUB model group; (C) DXL group; (D) BYJ group

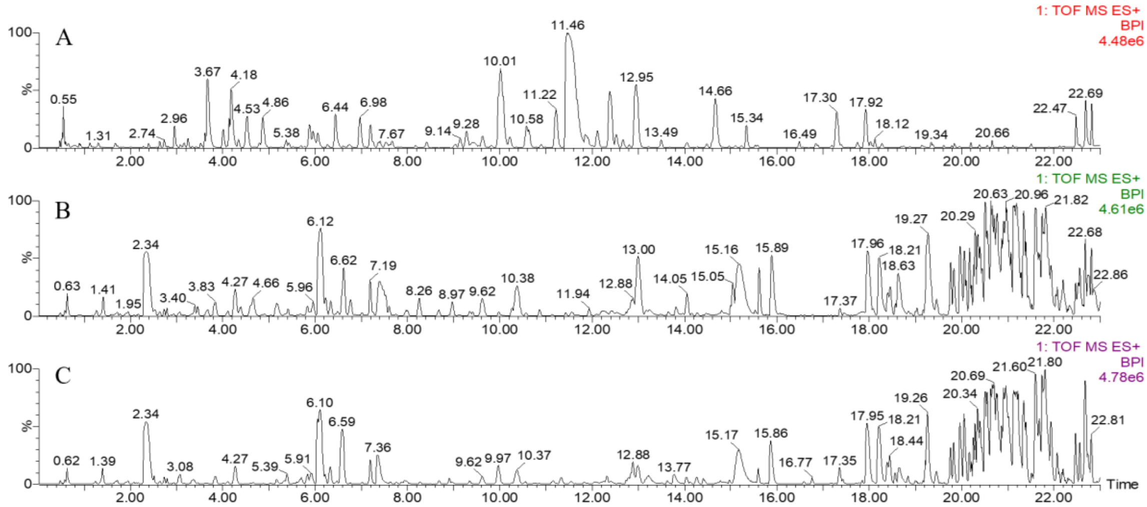

Figure S3. Chromatograms in serum pharmacochemistry analysis under positive ion mode. (A) BYJ *in vitro* sample; (B) AUB model serum sample; (C) Drug-containing serum sample.

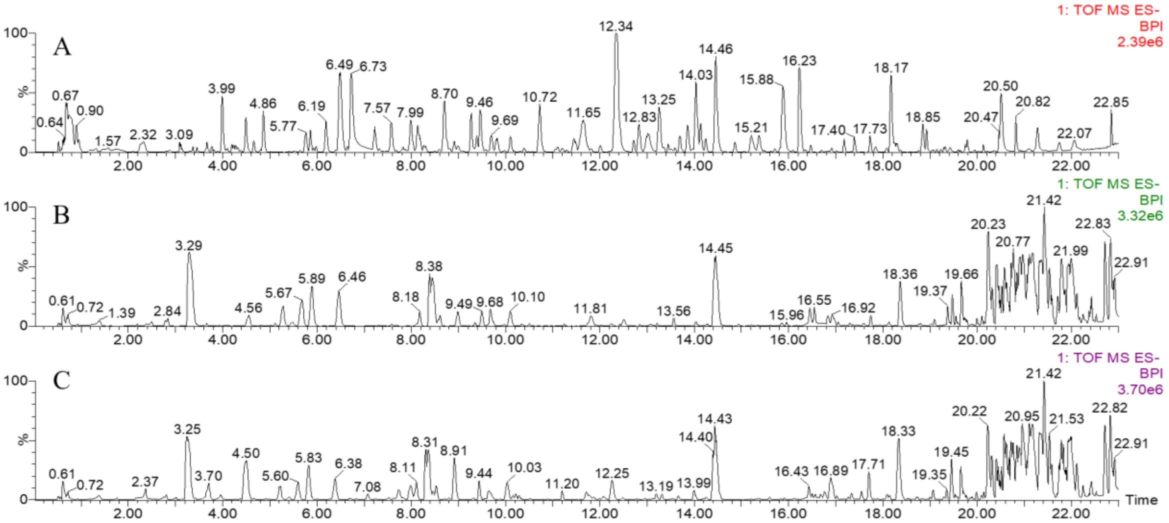

Figure S4. Chromatograms in serum pharmacochemistry analysis under negative ion mode.

(A) BYJ *in vitro* sample; (B) AUB model serum sample; (C) Drug-containing serum sample.

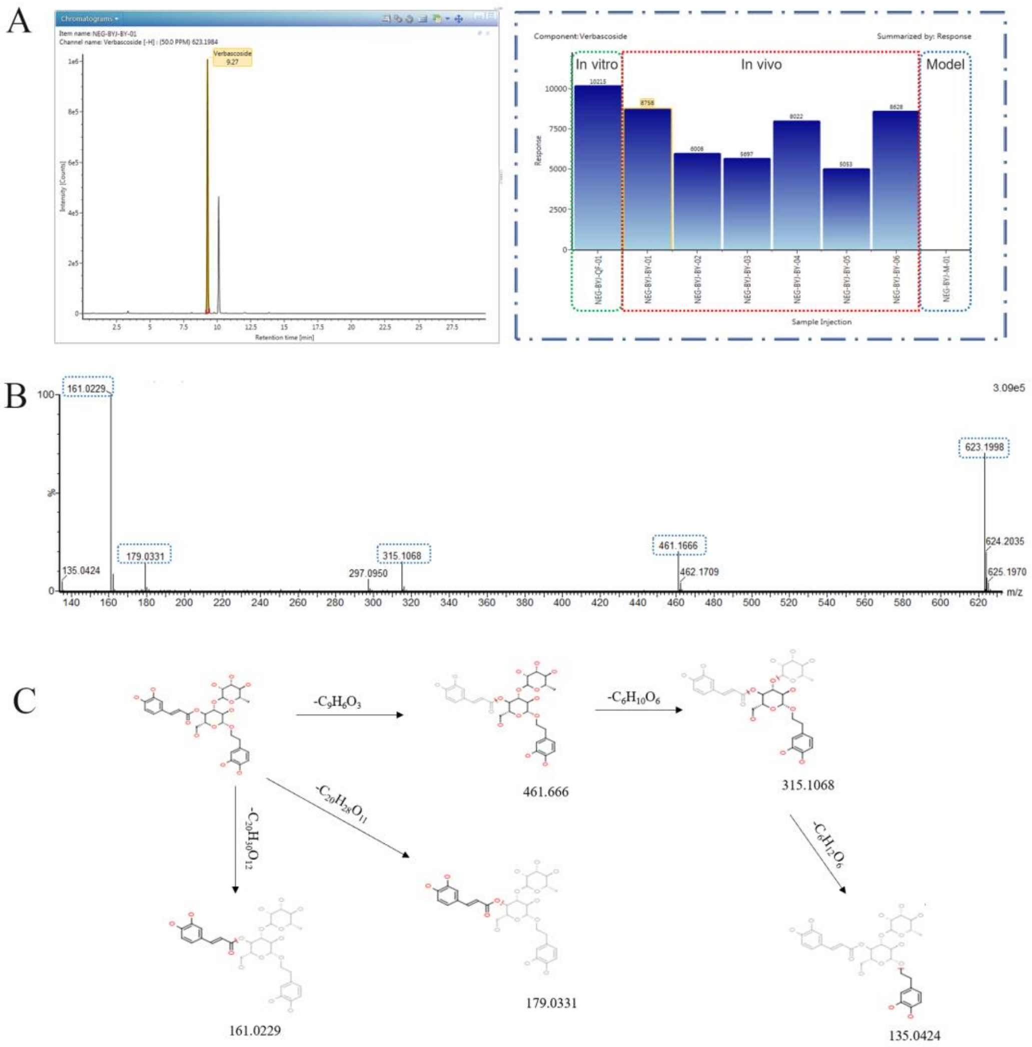

Figure S5. Correlation mass spectrum and cleavage pathway of verbascoside

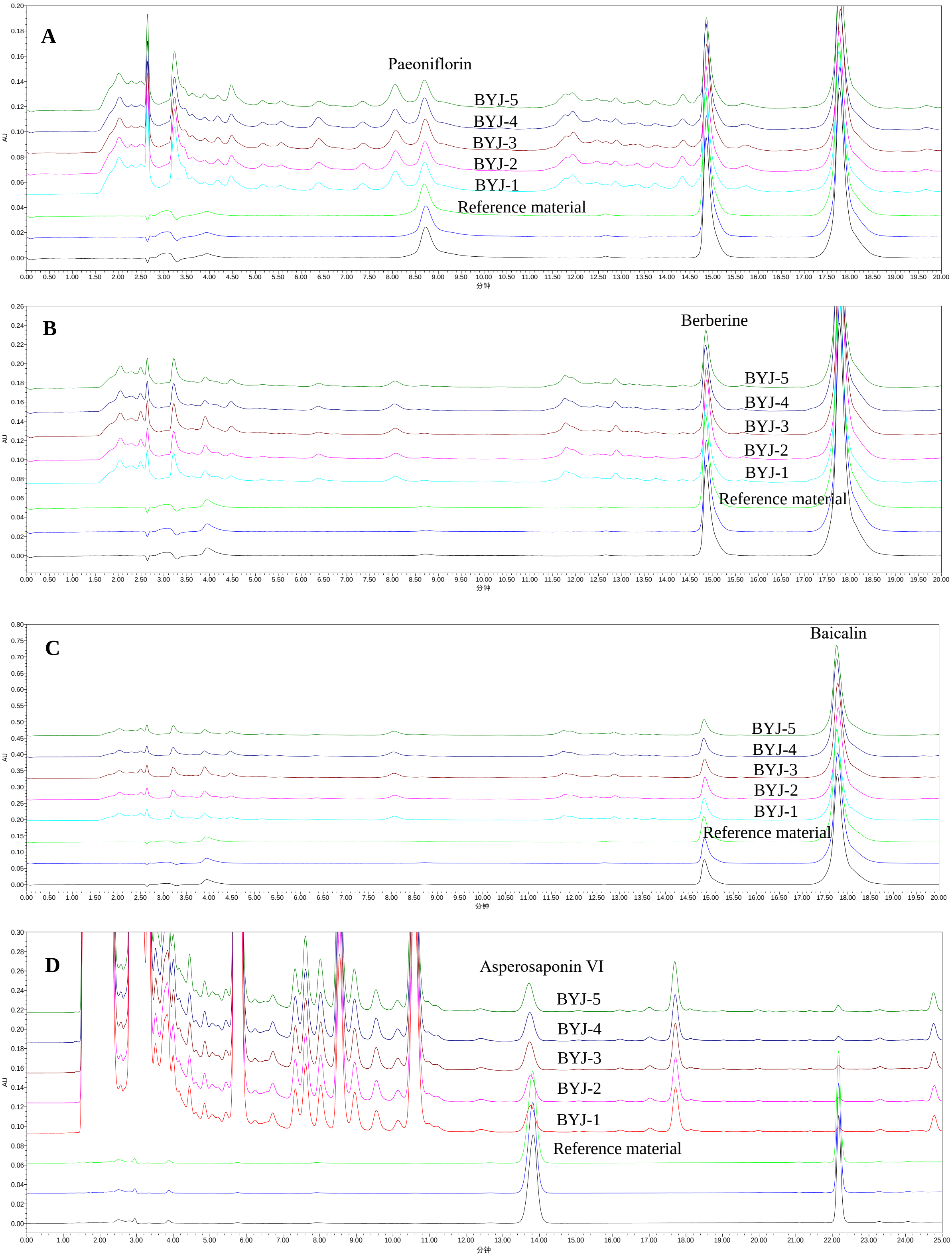
A
Paeoniflorin
BYJ-5
BYJ-4
BYJ-3
BYJ-2
BYJ-1
Reference material
AU
分钟
B
Berberine
BYJ-5
BYJ-4
BYJ-3
BYJ-2
BYJ-1
Reference material
AU
分钟
C
Baicalin
BYJ-5
BYJ-4
BYJ-3
BYJ-2
BYJ-1
Reference material
AU
分钟
D
Asperosaponin VI
BYJ-5
BYJ-4
BYJ-3
BYJ-2
BYJ-1
Reference material
AU
分钟

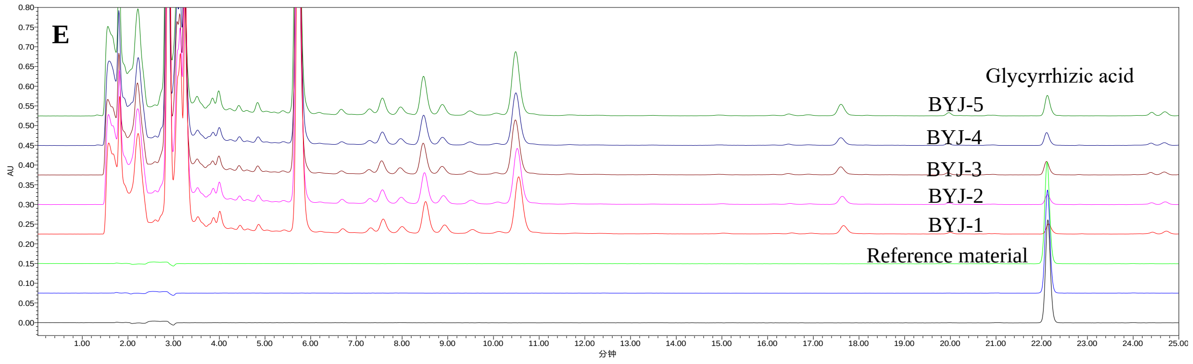

Figure S6. Content determination of BYJ

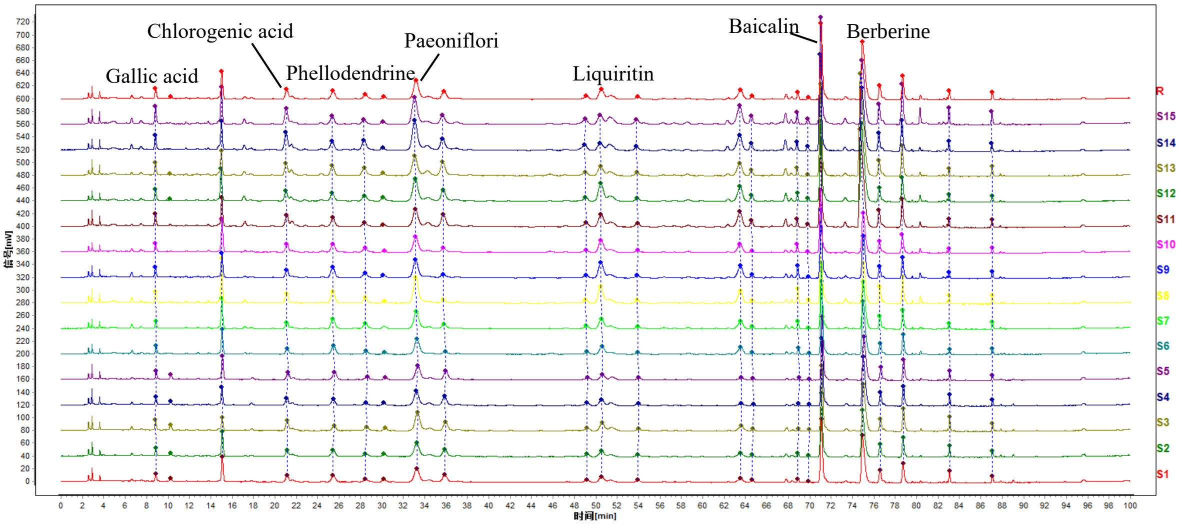

Figure S7. Fingerprint similarity of 15 batches of BYJ

Supplementary Tables

Table S1. Model parameter of OPLS-DA

OPLS-DA	R^2Y(cum)	Q^2(cum)	Intercept of R^2	Intercept of Q^2
POS	0.995	0.927	0.923	-0.242
Neg	0.986	0.904	0.946	-0.357

Table S2. Results of metabolic pathway analysis of AUB-related markers based on MetPA

NO.	Pathway name	Class	Total	Hits	Raw p	-log(p)	Impact
1	Arachidonic acid metabolism	Lipid metabolism	36	7	0.000005	5.2737	0.43392
2	Steroid hormone biosynthesis	Lipid metabolism	77	6	0.004586	2.3386	0.21318
3	Glycerophospholipid metabolism	Lipid metabolism	36	4	0.0061399	2.2118	0.30986
4	Tryptophan metabolism	Amino acid metabolism	41	4	0.0097951	2.009	0.19641
5	alpha-Linolenic acid metabolism	Lipid metabolism	13	2	0.02949	1.5303	0.33333
6	Retinol metabolism	Metabolism of cofactors and vitamins	16	2	0.04361	1.3604	0.22754
7	Citrate cycle (TCA cycle)	Carbohydrate metabolism	20	2	0.065517	1.1836	0.14894
8	Alanine, aspartate and glutamate metabolism	Amino acid metabolism	28	2	0.11745	0.93014	0.04808
9	D-Glutamine and D-glutamate metabolism	Amino acid metabolism	6	1	0.1211	0.91687	0
10	Purine metabolism	Nucleotide metabolism	66	3	0.16205	0.79034	0.01602
11	Biosynthesis of unsaturated fatty acids	Lipid metabolism	36	2	0.1766	0.753	0
12	Arginine biosynthesis	Amino acid metabolism	14	1	0.26066	0.58392	0
13	Glycosylphosphatidylinositol (GPI)-anchor biosynthesis	Glycan biosynthesis and metabolism	14	1	0.26066	0.58392	0.00399

NO.	Pathway name	Class	Total	Hits	Raw p	-log(p)	Impact
14	Butanoate metabolism	Carbohydrate metabolism	15	1	0.27652	0.55827	0
15	Glycerolipid metabolism	Lipid metabolism	16	1	0.29205	0.53455	0.01246
16	Ether lipid metabolism	Lipid metabolism	20	1	0.35099	0.4547	0
17	Pentose phosphate pathway	Carbohydrate metabolism	22	1	0.37865	0.42176	0.04712
18	Phosphatidylinositol signaling system	Signal transduction	28	1	0.45495	0.34203	0.00152
19	Glyoxylate and dicarboxylate metabolism	Carbohydrate metabolism	32	1	0.50069	0.30043	0.03175
20	Tyrosine metabolism	Amino acid metabolism	42	1	0.59936	0.22231	0.01751

Table S3. Identification of the components in serum after oral administration of BYJ solution

NO.	R_t (min)	Formula	Component	Neutral mass (Da)	Observed m/z	Mass error (mDa)	Ion mode	Fragment	Origin
1	1.90	$C_{15}H_{22}O_{10}$	Catalpol	362.1218	407.12	0.5	$[M-H-COOH]^-$	199/181/169	a,b
2	2.00	$C_7H_6O_5$	Gallic acid	170.0215	169.014	-0.3	$[M-H]^-$	125/107/97	c,g
3	2.52	$C_{20}H_{30}O_{12}$	Verbascoside-$C_9H_6O_3$(cleavage)	462.1722	463.1794	-1.6	$[M+H]^+$	286/255/184	a,b
4	2.61	$C_{16}H_{26}O_{10}$	Logaric acid (Loganic acid)+H_2	378.1532	377.1459	0.5	$[M-H]^-$	328/300/192	f
5	2.77	$C_{11}H_{12}O_7$	Catalpol-$C_6H_{10}O_5$(cleavage)+O-H_2+C_2H_2O	256.0592	255.0519	0.9	$[M-H]^-$	225/218/137	a,b
6	2.86	$C_9H_6O_3$	Verbascoside-$C_{20}H_{28}O_{12}$(cleavage)-H_2	162.0329	163.0401	1.2	$[M+H]^+$	146/128/120	a,b
7	2.95	$C_{15}H_{24}O_9$	Ajugol	348.14203	347.1342	0.5	$[M-H]^-$	329/167/149	a,b
8	3.09	$C_{27}H_{42}O_{20}$	Rehmannioside D	686.22694	731.2255	0.3	$[M-H-COOH]^-$	505/323/179	a,b
9	3.66	$C_{16}H_{24}O_{10}$	8-Epiloganic acid	376.13695	375.1291	0	$[M-H]^-$	213/169/151	a,b

NO.	R_t (min)	Formula	Component	Neutral mass (Da)	Observed m/z	Mass error (mDa)	Ion mode	Fragment	Origin
10	3.93	$C_{25}H_{20}NO_9$	Berberine-CHO(cleavage)+2x(-H_2)+$C_6H_8O_6$	478.1109	479.1182	-2.8	$[M+H]^+$	381/197/127	e
11	3.94	$C_{16}H_{24}O_{10}$	Loganic acid	376.1369	375.1291	0.6	$[M-H]^-$	213/169/151	f
12	4.11	$C_{16}H_{18}O_9$	Chlorogenic acid	354.09508	353.0873	0.1	$[M-H]^-$	191/179173	e,f
13	4.21	$C_{20}H_{24}NO_4$	Phellodendrine	342.1705	342.1725	0.7	M^+	192/177/237	e
14	4.34	$C_{16}H_{18}O_9$	Neochlorogenic acid	354.09508	353.0879	0	$[M-H]^-$	191/179/173	e,f
15	4.45	$C_{23}H_{28}O_{12}$	Oxypaeoniflorin	496.1581	495.1511	0.3	$[M-H]^-$	465/333/165	c
16	4.86	$C_9H_8O_4$	Caffeic acid	180.04226	179.035	0	$[M-H]^-$	161/135	a,b
17	5.36	$C_{25}H_{26}NO_{10}$	Berberine-CH(cleavage)+H_2+$C_6H_8O_6$	500.1515	500.1515	-4.3	M^+	452/324/201	e
18	5.71	$C_{21}H_{20}O_{10}$	Liquiritigenin+$C_6H_8O_6$	432.1058	433.1131	0.1	$[M+H]^+$	257/211/137	h
19	5.92	$C_{15}H_{12}O_4$	Liquiritigenin	256.07356	255.0659	-0.4	$[M-H]^-$	135/119	h
20	5.94	$C_{17}H_{26}O_{10}$	Loganin	390.1526	435.1506	-0.2	$[M-H-COOH]^-$	343/195/181	f
21	6.19	$C_{23}H_{28}O_{11}$	Albiflorin	480.1582	525.1582	0.4	$[M-H-COOH]^-$	449/327/165	c
22	6.29	$C_{17}H_{20}O_9$	4-O-Feruloylquinic acid	368.1107	367.1029	1	$[M-H]^-$	173/193/191	e
23	6.37	$C_{17}H_{18}O_8S$	Paeoniflorin-$C_6H_{10}O_6$(cleavage)+SO_3	382.068	381.0607	-4.3	$[M-H]^-$	265/175/113	c
24	6.73	$C_{23}H_{28}O_{11}$	Paeoniflorin	480.1582	525.1582	0	$[M-H-COOH]^-$	449/327/165	c
25	7.08	$C_{15}H_{10}O_7$	QuerceTIN	302.0427	301.0348	0.3	$[M-H]^-$	274/151	h
26	7.29	$C_{23}H_{26}O_{14}S$	Albiflorin-H_2+SO_3	558.0999	559.1071	-4.4	$[M+H]^+$	507/331/201	c
27	7.34	$C_{17}H_{20}O_9$	5-O-Feruloylquinic acid	368.1107	367.1029	0.5	$[M-H]^-$	191/173/134	e
28	7.63	$C_{21}H_{20}O_{12}$	Baicalin+O+H_2	464.0958	463.0885	0.3	$[M-H]^-$	366/312/287	d
29	7.90	$C_{21}H_{22}O_9$	Liquiritin	418.12638	417.1193	0.2	$[M-H]^-$	255/135/119	h
30	8.38	$C_{20}H_{26}O_{14}S$	Verbascoside-$C_9H_6O_4$(cleavage)+2x(-H_2)+SO_3	522.1022	521.0949	-2	$[M-H]^-$	475/451/323	a,b

NO.	R_t (min)	Formula	Component	Neutral mass (Da)	Observed m/z	Mass error (mDa)	Ion mode	Fragment	Origin
31	8.67	$C_9H_{10}O_2$	Paeoniflorin-$C_{16}H_{22}O_{10}$(cleavage)+H_2+C_2H_2O	150.0693	149.062	1.1	$[M-H]^-$	137/113/93	c
32	9.18	$C_{21}H_{25}NO_4$	Menisperine	356.1862	356.188	0.5	M^+	311/279/248	e
33	9.27	$C_{29}H_{36}O_{15}$	Verbascoside	624.20542	623.1984	0.3	$[M-H]^-$	315/135/161	a,b
34	9.54	$C_{20}H_{19}NO_4$	Jatrorrhizine	338.1401	338.1382	0.3	M^+	323/294	e
35	9.98	$C_{21}H_{18}O_{11}$	Baicalin	446.08491	447.0927	0.7	$[M+H]^+$	423/407/396	d
36	10.13	$C_{27}H_{28}O_{16}$	Liquiritin+O-H_2+$C_6H_8O_6$	608.1383	607.131	0.6	$[M-H]^-$	431/269/187	h
37	10.83	$C_{23}H_{22}O_{13}$	Baicalein+H_2	272.0692	273.0765	0.7	$[M+H]^+$	259/177/131	d
38	11.44	$C_{17}H_{14}O_8$	Viscidulin III	346.06887	345.0621	0.5	$[M-H]^-$	345/330/315	d
39	11.46	$C_{20}H_{17}NO_4$	Berberine	336.1236	336.1236	0.1	M^+	321/292	e
40	12.90	$C_{21}H_{20}O_{11}$	Cynaroside	448.10056	493.0982	-0.6	$[M-H-COOH]^-$	285/151	d
41	12.88	$C_{22}H_{20}O_{11}$	Wogonoside	460.10056	461.106	0.5	$[M+H]^+$	285/270/242	d
42	13.37	$C_{23}H_{34}O_{18}$	Rehmannioside D-$C_6H_{10}O_5$(cleavage)+2x(+O)+C_2H_2O	598.1725	599.1797	-2.1	$[M+H]^+$	507/287/279	a,b
43	13.80	$C_{15}H_{10}O_4$	Chrysin	254.05791	253.0506	0	$[M-H]^-$	299/253	d
44	14.22	$C_{31}H_{40}O_{15}$	Isomartynoside	652.23672	651.2296	0.2	$[M-H]^-$	615/506/475	a,b
45	14.37	$C_{42}H_{58}O_{19}S$	Glycyrrhizic acid+2x([M-H]-2)+SO_3	898.3306	899.3379	1.5	$[M+H]^+$	581/463/331	h
46	14.66	$C_{15}H_{10}O_5$	Baicalein	270.0534	271.0607	0.6	$[M+H]^+$	271/253/243	d
47	14.87	$C_{20}H_{16}O_6$	Semilicoisoflavone B	352.0954	353.1027	0.7	$[M+H]^+$	311/325/299	h
48	15.12	$C_{47}H_{78}O_{18}$	Asperosaponin VI-$C_{12}H_{20}O_{10}$(cleavage)+O+H_2+SO_3	702.3665	703.3738	1.6	$[M+H]^+$	639/314/289	f
49	15.53	$C_{16}H_{12}O_4$	Formononetin	268.074	269.0813	0.5	$[M+H]^+$	254/238	h
50	16.24	$C_{47}H_{76}O_{18}$	Asperosaponin VI	928.5025	927.4953	0.2	$[M-H]^-$	603/323	f
51	16.56	$C_{23}H_{26}O_{12}$	Albiflorin+O-H_2	494.1435	493.1362	1.1	$[M-H]^-$	317/287/227	c

NO.	R_t (min)	Formula	Component	Neutral mass (Da)	Observed m/z	Mass error (mDa)	Ion mode	Fragment	Origin
52	17.19	$C_{15}H_{16}O_3$	Batatasin III	244.10994	243.1026	0	$[M-H]^-$	277/183/136	g
53	17.30	$C_{16}H_{12}O_5$	Wogonin	284.06847	285.0771	0.1	$[M+H]^+$	270/242/213	d
54	17.72	$C_{17}H_{14}O_6$	Baicalein+H_2+C_2H_2O	314.0804	315.0877	1.3	$[M+H]^+$	285/257/235	d
55	18.19	$C_{42}H_{62}O_{16}$	Glycyrrhizic acid	822.40379	821.3971	0.5	$[M-H]^-$	759/645/351	h
56	18.33	$C_{16}H_{12}O_5$	Oroxylin A	284.0685	283.0606	0.4	$[M-H]^-$	268/265/225	d
57	19.05	$C_{21}H_{20}O_5$	Gancaonin G	352.1311	353.1389	-0.2	$[M+H]^+$	193/213/167	h
58	19.32	$C_{26}H_{30}O_7$	Obacunone	454.19915	455.2070	0.6	$[M+H]^+$	437/409/436	e
59	21.90	$C_{30}H_{46}O_4$	Glycyrrhetinic acid	470.3396	471.3474	0.1	$[M+H]^+$	425/407/235	h

Note: a: *Rehmannia glutinosa* Libosch., b: Radix *Rehmanniae Praeparata*, c: *Paeonia lactiflora* Pall., d: *Scutellaria baicalensis* Georgi, e: *Phellodendron chinense* Schneid., f: *Dipsacus asper* Wall. ex Henry, g: *Dioscorea opposita* Thunb., h: *Glycyrrhiza uralensis* Fisch.

Table S4. Correlation analysis results of effective components and biomarkers

No.	Constituents	Relevant quantity	Source	No.	Constituents	Relevant quantity	Source
*1	Catalpol	5	a,b	31	Paeoniflorin-$C_{16}H_{22}O_{10}$(cleavage)+H_2+C_2H_2O	2	c
*2	Gallic acid	5	c,g	32	Menisperine	1	e
3	Verbascoside-$C_9H_6O_3$(cleavage)	2	a,b	33	Verbascoside	1	a,b
4	Logaric acid (Loganic acid)+H_2	0	f	34	Jatrorrhizine	2	e
5	Catalpol-$C_6H_{10}O_5$(cleavage)+O-H_2+C_2H_2O	2	a,b	*35	Baicalin	6	d
6	Verbascoside-$C_{20}H_{28}O_{12}$(cleavage)-H_2	4	a,b	*36	Liquiritin+O-H_2+$C_6H_8O_6$	5	h
7	Ajugol	2	a,b	37	Baicalein+H_2	1	d

No.	Constituents	Relevant quantity	Source	No.	Constituents	Relevant quantity	Source
*8	Rehmannioside D	5	a,b	38	Viscidulin III	2	d
9	8-Epiloganic acid	0	a,b	*39	Berberine	7	e
10	Berberine-CHO(cleavage)+2x(-H_2)+$C_6H_8O_6$	2	e	40	Cynaroside	3	d
11	Loganic acid	2	f	41	Wogonoside	1	d
*12	Chlorogenic acid	6	e,f	42	Rehmannioside D-$C_6H_{10}O_5$(cleavage)+2x(+O)+C_2H_2O	3	a,b
*13	Phellodendrine	6	e	43	Chrysin	3	d
14	Neochlorogenic acid	2	e,f	44	Isomartynoside	1	a,b
15	Oxypaeoniflorin	3	c	45	Glycyrrhizic acid+2x([M-H]-2)+SO_3	3	h
16	Caffeic acid	2	a,b	46	Baicalein	2	d
17	Berberine-CH(cleavage)+H_2+$C_6H_8O_6$	0	e	47	Semilicoisoflavone B	0	h
18	Liquiritigenin+$C_6H_8O_6$	1	h	48	Asperosaponin VI-$C_{12}H_{20}O_{10}$(cleavage)+O+H_2+SO_3	4	f
19	Liquiritigenin	2	h	49	Formononetin	1	h
20	Loganin	2	f	*50	Asperosaponin VI	5	f
21	Albiflorin	3	c	51	Albiflorin+O-H_2	3	c
22	4-O-Feruloylquinic acid	3	e	52	Batatasin III	1	g
23	Paeoniflorin-$C_6H_{10}O_6$(cleavage)+SO_3	2	c	53	Wogonin	1	d
*24	Paeoniflorin	7	c	54	Baicalein+H_2+C_2H_2O	2	d
25	QuerceTIN	1	h	55	Glycyrrhizic acid	1	h
26	Albiflorin-H_2+SO_3	3	c	56	Oroxylin A	1	d
27	5-O-Feruloylquinic acid	2	e	57	Gancaonin G	3	h
*28	Baicalin+O+H_2	5	d	58	Obacunone	2	e
*29	Liquiritin	6	h	*59	Glycyrrhetinic acid	5	h

No.	Constituents	Relevant quantity	Source	No.	Constituents	Relevant quantity	Source
30	Verbascoside-$C_9H_6O_4$(cleavage)+2x(-H_2)+SO_3	3	a,b				

Note: a: *Rehmannia glutinosa* Libosch., b: Radix *Rehmanniae Praeparata,* c: *Paeonia lactiflora* Pall., d: *Scutellaria baicalensis* Georgi, e: *Phellodendron chinense* Schneid., f: *Dipsacus asper* Wall. ex Henry, g: *Dioscorea opposita* Thunb., h: *Glycyrrhiza uralensis* Fisch. , "*": Correlation quantity>5.

Table S5. Content determination results in five batches of BYJ

Component	Batch number	Content（mg/g）	Average content（mg/g）	RSD（%）
Paeoniflorin	1	8.386		
	2	8.670		
	3	8.559	8.558	1.23
	4	8.579		
	5	8.596		
Berberine	1	5.338		
	2	5.412		
	3	5.567	5.420	1.8
	4	5.328		
	5	5.453		
Baicalin	1	28.180		
	2	27.900		
	3	27.800	28.098	1.3
	4	27.910		
	5	28.700		

Asperosaponin VI	1	2.891		
	2	2.953		
	3	2.932	2.943	1.26
	4	2.944		
	5	2.994		
Glycyrrhizic acid	1	3.169		
	2	3.287		
	3	3.288	3.236	1.66
	4	3.195		
	5	3.241		

Table S6. Fingerprint similarity of 15 batches of BYJ

Batch number	S1	S2	S3	S4	S5	S6	S7	S8	S9	S10	S11	S12	S13	S14	S15
similarity (control)	0.99	0.99	0.99	0.99	0.99	0.99	0.99	0.94	0.97	0.98	0.96	0.98	0.96	0.98	0.98
